# Overcoming tumor microenvironment barriers: transformable and bioinspired nanomedicine strategies for deep tumor penetration

**DOI:** 10.1186/s12951-026-04028-7

**Published:** 2026-01-30

**Authors:** Jiabao Sheng, Weisi Yuan, Mingjun Zhang, Ze Chen, Yanfei Qi, Yinan Zhao, Shubiao Zhang

**Affiliations:** 1 Key Laboratory of Biotechnology and Bioresources Utilization of Ministry of Education, Dalian Minzu University, Dalian, 116600 China; 2 Heart Research Institute, The University of Sydney, Newtown, NSW 2042 Australia

**Keywords:** Tumor microenvironment, Nanomedicine delivery, Intratumoral penetration, Size and charge transformation, Barrier modulation, Bioinspired and multistage systems

## Abstract

Solid tumors pose a spatial “delivery-at-depth” bottleneck: therapeutics that reach tumors often remain sequestered near vessels and fail to distribute uniformly into tumor cores. This limitation arises from heterogeneous perfusion, elevated interstitial fluid pressure, and dense extracellular matrix, which together restrict convection–diffusion balance and amplify binding-site barriers. We organize transformable and bioinspired nanomedicines using a barrier-centric lens and summarize five strategy families to deepen and homogenize intratumoral transport: (i) stimuli-responsive size/charge switching, (ii) microenvironment remodeling to restore perfusion and decompress stroma, (iii) ligand-guided transcytosis and CendR pathway engagement, (iv) cell-based and biomimetic vectors leveraging homing and immune evasion, and (v) multistage designs that sequence priming, switching, and payload activation. We compare representative systems by trigger specificity, activation timing, affinity tuning, and corona susceptibility, and highlight recurring failure modes including stimulus heterogeneity, premature/off-target activation, and escalating chemistry–manufacturing–controls burdens with added components. We conclude with translational priorities: couple barrier priming with a single well-characterized switching event, favor moderated or activatable affinity to avoid perivascular trapping, and validate spatial gains using standardized intratumoral distribution metrics linked to therapeutic endpoints.

**Graphical Abstract:**

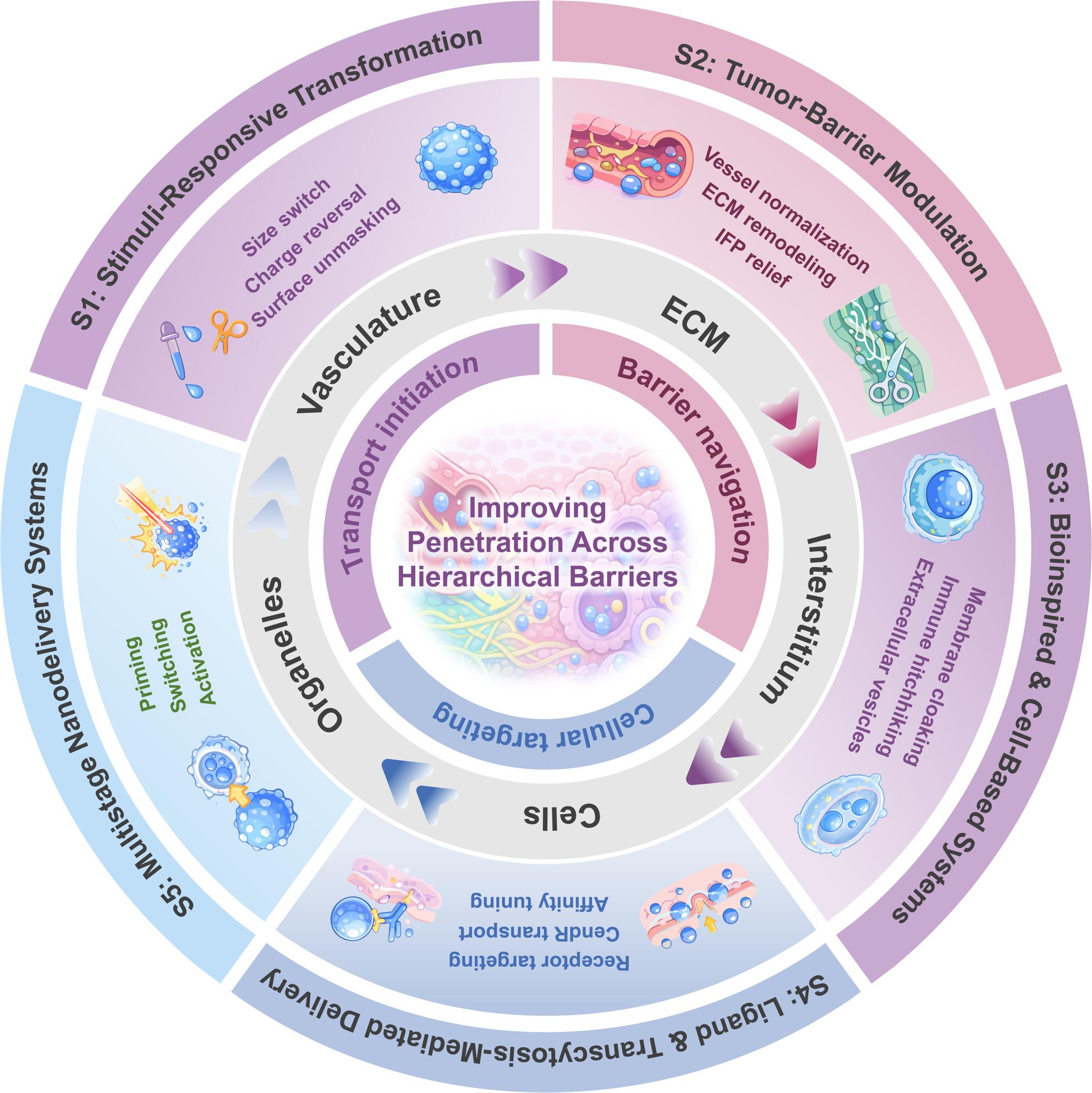

## Background

Tumors remain among the most formidable challenges to global health [1]. Despite advances in chemical drugs, antibody therapeutics, and gene therapies, the overall prognosis for patients with solid tumors remains unsatisfactory. A major obstacle lies in the inefficient intratumoral delivery of therapeutics [2]. Systemically administered drugs are subject to rapid clearance, limited tumor accumulation, and most critically, poor tissue penetration. These limitations substantially diminish therapeutic efficacy, especially in dense and heterogeneous solid tumors [3]. Nanotechnology-based drug delivery systems offer new opportunities to improve pharmacokinetics, enable site-specific targeting, and enhance drug stability in circulation [4]. Several nanomedicines, such as liposomal doxorubicin (DOX) and albumin-bound paclitaxel, have been approved for clinical use, with benefits primarily in reducing systemic toxicity. However, their therapeutic superiority over free drugs has been modest, often due to insufficient distribution within the tumor tissues [5]. Effective delivery to solid tumors requires success across five sequential steps: systemic circulation, tumor accumulation, deep tissue penetration, cellular internalization, and controlled drug release [6]. Among these, intratumoral penetration remains the most critical and limiting factor, particularly for nanoparticle-based systems. Penetration efficiency is influenced not only by the physicochemical properties of nanocarriers, such as size, charge, and flexibility, but also by the unique structural and physiological characteristics of the tumor microenvironment (TME) [7]. Compared with normal tissues, tumors exhibit a constellation of abnormal features collectively referred to as the TME, including disorganized and hyperpermeable vasculature, dense extracellular matrix (ECM), elevated interstitial fluid pressure (IFP), aberrant enzyme expression, acidic pH, and hypoxic regions. Together, these factors form multilayered barriers that restrict uniform drug distribution and deep infiltration. In addition to imposing physical transport barriers, stromal cells can actively drive malignant progression and therapy resistance. For example, lung cancer-associated mesenchymal stem cells promote autophagy and epithelial-mesenchymal transition through HIF-1α-dependent signaling, thereby enhancing invasion and chemoresistance and underscoring that tumor-microenvironment remodeling is tightly coupled to drug response as well as penetration [8]. Interestingly, what appear to be barriers can also be exploited as triggers: local acidity and enzymatic activity can induce size shrinkage and charge reversal; hypoxia and redox gradients can open transcytotic pathways and expose latent ligands. Through such mechanisms, these cues can be harnessed to invert biological filters and propel nanomedicines beyond perivascular regions into tumor cores [9].

**Fig. 1:**
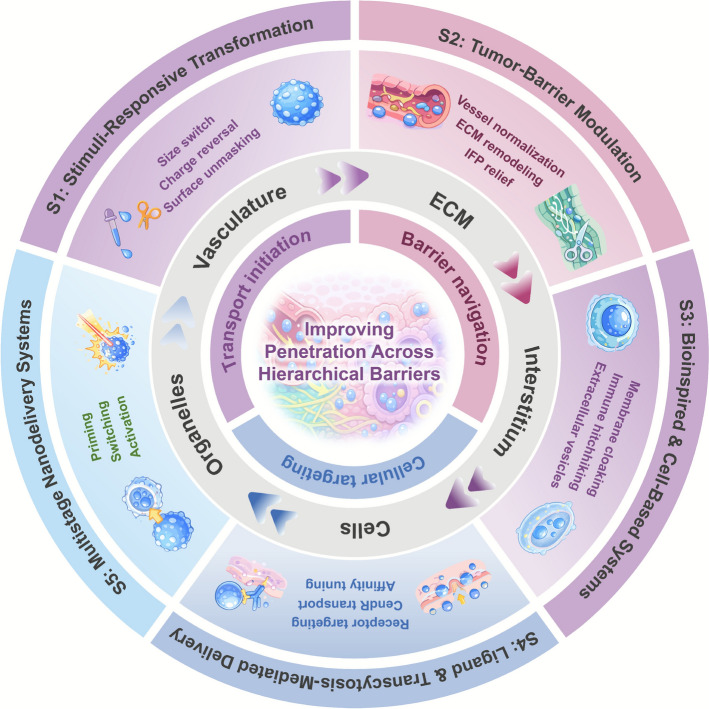
Modular, barrier-centric map of strategies for deep tumor penetration. The outer ring summarizes five strategy families (S1–S5). The middle ring indicates the dominant barrier level targeted (vasculature, ECM/IFP, interstitium, and cellular barriers). Inner icons denote representative triggers (e.g., pH, enzymes, redox, hypoxia, shear) and transformation outputs (e.g., size/charge/ligand exposure) used across the five families

Beyond mechanistic elegance, however, it is critical to assess whether these designs truly enhance intratumoral penetration and therapeutic performance in vivo [10]. To date, most penetration claims have been established in subcutaneous or orthotopic xenografts and relatively simple 3D spheroids, which only partially recapitulate patient-level heterogeneity in perfusion, stromal architecture and immune contexture. Emerging patient-derived organoids (PDOs), co-culture spheroids and microfluidic tumor-on-a-chip platforms offer more faithful TME architectures and spatiotemporal gradients, and are thus particularly well suited for benchmarking “deep penetration” performance and validating model-informed design rules [11]. In this review, we classify strategies into five complementary directions: (i) TME remodeling/barrier relief, (ii) size and charge transformation, (iii) ligand- and ligand-guided transcytosis driven by the C-end Rule (CendR) motif through neuropilin-1 (NRP1)-guided transcytosis, (iv) cell-based and bioinspired carriers (including cell-penetrating peptide (CPP) modules), and (v) multistage cascade systems (Fig. 1). We evaluate these approaches in terms of intratumoral distribution, tissue coverage, and penetration depth, together with their in vivo therapeutic outcomes. Furthermore, we compare traditional and smart delivery platforms across tumor models, highlight key design limitations, and propose model-informed solutions to guide rational design and clinical translation [12–14].

Compared with previous reviews that focus separately on transformable nanomedicine, TME-triggered delivery, or multistage carrier design, our article aims to fill a transport- and barrier-centered gap [15]. Earlier overviews have typically organized systems by material class or by a single trigger, but they seldom cross-compare how different design logics perform against specific tumor barriers or how deeply they penetrate in three-dimensional models and in vivo [16]. Here we instead map five strategy families, size-shrinkable and charge-reversible nanocarriers, vascular/ECM/IFP-remodeling platforms, ligand- and CendR-mediated transcytosis systems, cell-based and other bioinspired vectors (including CPP-assisted designs), and multistage cascades onto a common framework that is explicitly defined by intratumoral transport steps and measurable penetration readouts. We further couple this cross-classification with a discussion of manufacturability and safety constraints that are often only briefly mentioned in prior work, with the goal of providing practical, clinically oriented design rules for achieving deep and homogeneous tumor penetration. In this Review, we synthesize five penetration-enabling strategy families (S1-S5), spanning physicochemical switching, microenvironment remodeling, transcytosis/CendR engagement, bioinspired vectors, and multistage sequencing. Figure 1 provides a modular map that links each family to the dominant barrier level(s) targeted, enabling rapid navigation between mechanism, barrier context, and representative design patterns.

## Size and charge transformation

**Fig. 2:**
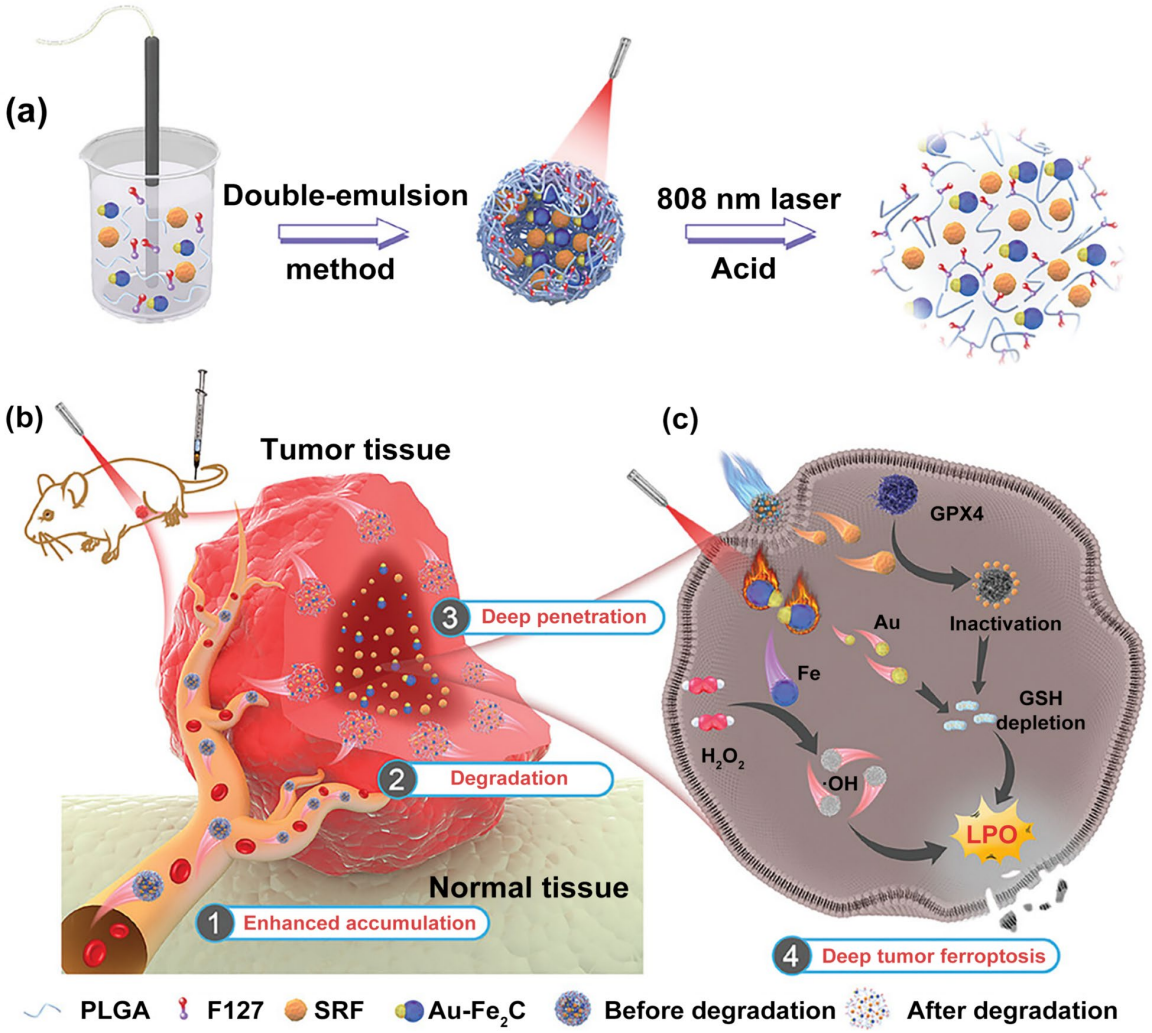
Size switchable Au-Fe_2_C nanocapsules enable cascade delivery in the tumor microenvironment. Acidic or mild thermal cues degrade the shell and release ultrasmall Au-Fe_2_C cores for deep penetration, coupled with glutathione depletion, Fenton chemistry, and sorafenib-mediated GPX4 inhibition to drive ferroptosis. *Adapted with permission from Wang, J.; Fang, Z.; Zhao, C.; *et al*., Advanced Materials 36 (2024) 2,307,006, * 10.1002/adma.202307006*.* © 2023 Wiley–VCH GmbH. Permission under Wiley License No. 6121350053588. Changes were made. [21]

In three dimensional tumor models, recent work using well-defined gold nanoparticles highlights the multi-parameter dependence of penetration on both physicochemical properties and material composition. In the same study, Cybulski et al. further demonstrated that positively charged AuNR(+) underwent complete charge inversion in serum-containing medium due to the adsorption of a predominantly anionic protein corona, which reduced close contact with cell membranes and limited penetration into inner spheroid layers [17]. Recent corona-focused analyses likewise emphasize that dynamic corona formation can substantially shift the zeta potential of nanoparticles and sterically shield pH- or redox-sensitive moieties [18]. As a result, transformation kinetics and activation windows calibrated in protein-free buffers may differ markedly from those in plasma or interstitial fluid, underscoring the need to validate size- and charge-switching behavior under physiologically relevant protein concentrations [18]. They observed that larger nanospheres showed higher overall accumulation but shallower penetration, whereas smaller nanospheres and negatively charged particles could reach inner spheroid layers, and rod-shaped particles generally displayed more limited penetration than spherical ones [17]. Despite these insights, efficient intratumoral transport remains a major hurdle in solid tumor therapy. Nanoparticles larger than 100 nm are more stable in circulation, with prolonged blood half-life, but face significant challenges in penetrating the dense ECM and IFP of tumors. Conversely, smaller particles (< 30 nm) diffuse more readily into deep tumor regions but are quickly cleared from circulation and may cause off-target effects [19]. Therefore, a key design challenge is balancing systemic stability with efficient tumor penetration. To address this issue, researchers have developed transformable nanocarriers whose size or surface charge can dynamically respond to specific TME stimuli, such as pH, enzymes, or GSH [20]. These systems typically adopt a “long-circulation, local-shrinkage” strategy, where large particles contract or disassemble at the tumor site, improving extravasation and deep tissue diffusion. This approach effectively balances systemic stability with deep intratumoral penetration (Fig. 2). Beyond these widely used pH- and GSH/redox-triggered systems, several size- and charge-transformable platforms have also been engineered to respond to enzyme activity, hypoxia, or externally applied cues such as mild hyperthermia or ultrasound, as illustrated in the following examples. Within the framework in Fig. 1, these systems are mainly triggered by pH, redox and enzymatic cues, undergo size shrinkage and/or charge reversal, act predominantly at the ECM/interstitial and cellular levels, and are typically activated after a brief priming phase.

### Size shrinkable systems

Across acid or redox biased shrinkers, two recurrent behaviors emerge. First, modest contraction, approximately twofold, already reduces steric hindrance and improves interstitial diffusion. Second, ultra-shrinkage to sub-10 ~ 20 nm fragments is most effective at overcoming ECM and IFP bottlenecks in stroma-rich tumors [22]. Representative designs span from tens-of-nanometers carriers that contract to the high-teens, to protease-addressable constructs that unmask ultrasmall building blocks, and to noncovalent assemblies that disassemble into approximately 10 nm entities under acidic and GSH-rich conditions [22]. In comparative terms, acid/GSH shrinkers emphasize interstitial passage and retention with relatively simple chemistries [22, 23], whereas protease-directed ultra-shrinkers are especially potent in desmoplastic matrices that overexpress MMPs, achieving deeper spread at the cost of more elaborate trigger design.

Because activated fragments are often neutral to mildly negative, diffusion gains can outpace cellular entry, which encourages coupling shrinkage with a second lever such as latent cation exposure or ligand unmasking so that transport is not achieved at the expense of uptake [22, 24]. In the extreme, ultra-shrinkage to the sub-10 nm regime raises a second-order trade-off between *depth* and *residence*. Very small fragments reduce steric hindrance and can traverse dense ECM more readily, yet they also lose multivalency and perivascular “trapping” effects that support cell/tissue retention, so the fraction achieving productive cellular association may decrease even when spatial distribution becomes more homogeneous. In addition, when the activated entities approach the ultrasmall range, concentration gradients can drive partial back-diffusion toward vessels, and systemic elimination can accelerate once fragments re-enter circulation, collectively increasing the risk of intratumoral washout. These considerations suggest that the most useful design space is often not “as small as possible’ but rather ‘small enough to move, yet large/interactive enough to stay”, motivating staged architectures that diffuse first and capture later. For example, (i) delayed exposure of mild cationic patches to increase membrane affinity at depth, (ii) ligand unmasking to restore receptor-mediated binding/internalization, (iii) microenvironment-triggered re-assembly or matrix anchoring to locally trap fragments, or (iv) deep-site drug release/prodrug activation so that pharmacodynamic gain is preserved even if carrier fragments are cleared [22, 24]. When shrinkers are combined with prior microenvironment priming, for example brief courses that lower IFP or soften ECM, the same activated size window, about 20–50 nm, tends to produce more uniform coverage than without priming.

### Charge reversal systems

Charge reversal strategies have been developed to improve cellular internalization and interstitial transport. Hu et al. designed hyaluronidase (HAase)-responsive tellurium/quaternary ammonium polycarbonate nanoparticles coated with hyaluronic acid, which mask their cationic core during circulation (*ζ* ~ −27 mV, 160 nm). Upon HAase digestion in the tumor bed, the shell is removed, shrinking the particles to approximately 130 nm and reversing the surface charge to positive. This process enhances adsorption-mediated transcytosis, allowing uniform penetration through 3.6 mm thick 2,3-Dimethylmaleic Anhydride (DMMA) xenografts and achieving potent tumor inhibition via mitochondrial cisplatin release with negligible systemic toxicity compared with the control groups [25]. Wan et al. reviewed β‑carboxylic amide-based DMMA shields, which remain stealthy in circulation but hydrolyze under acidic conditions to expose cationic surfaces, facilitating higher uptake while highlighting challenges in stability and manufacturability [26]. Acid-activated Met@BF nanohybrids change zeta potential from −16.6 mV to + 18.5 mV at pH 6.0, penetrate melanoma spheroids by about 60 µm from the periphery toward the avascular core, with ultrasound-driven BaTiO₃ piezocatalysis, produce near-complete control of primary and metastatic disease in vivo relative to free-drug control arms [27]. Compared with shrink-only designs, these systems increase cell engagement and adsorption-mediated transcytosis, elevating uptake where membrane passage is rate-limiting. Typical zeta-potential shifts, roughly from negative twenties to positive tens, are sufficient to boost internalization without extreme cationic densities that compromise safety. Since charge reversal does not inherently reduce hydrodynamic size, diffusion through dense stroma can remain the bottleneck when activated diameters exceed about 50 nm; pairing partial downsizing with charge programming or introducing short, well-tolerated priming that preserves perfusion can mitigate this constraint [25]. Dose and timing are critical, because excessive or prolonged anti-angiogenic exposure may reduce perfusion and counteract delivery, whereas short regimens can transiently enhance transport and thereby augment charge-reversal gains.

### Dual-transform systems

Dual-transformers integrate the strengths of both classes by staging the transitions: shrink first to lower extracellular resistance, then expose cationic or ligand interfaces to secure uptake at depth. Two sequencing archetypes have proven useful. First, pH followed by enzyme or GSH cascades, in which mild acidification initiates partial downsizing or loosens the corona before a second stimulus exposes cationic or targeting domains [28]. As discussed above, however, the formation and remodeling of a protein corona in vivo may delay or dampen these sequential switches, making it essential to benchmark dual-transform systems under realistic protein conditions rather than only in simple buffers. Legumain- or GSH-activated constructs first shrink and then unmask mildly cationic surfaces, yielding small entities with improved spread and capture [29]. Second, depot followed by regeneration strategies, where amphiphiles form small, blood-stable particles that precipitate at pH about 6.5 to create a local depot and subsequently hydrolyze to regenerate tens-of-nanometers, folate-targeted cationic spheres for sustained deep delivery [30]. Aligning the sequence of transformations with the expected perfusion window, for example ensuring that contraction precedes cation exposure while perfusion is still adequate, further improves spatial uniformity and pharmacodynamics.

As summarized in Table 1, most transformable nanocarriers are calibrated in well-controlled rodent models or 3D spheroids with relatively uniform stimuli, whereas human tumors display pronounced microregional heterogeneity in extracellular pH, enzyme activity and hypoxia. Clinical imaging and microelectrode studies indicate that the bulk extracellular pH of many solid tumors often remains around 6.8–7.1, with only patchy acidic niches falling below 6.7, so pH-triggered shrinkers or charge-reversal systems tuned to sharp transitions at pH 6.5–6.8 may transform efficiently in small, severely hypoxic regions but remain inactive in large portions of the lesion [31, 32]. Likewise, MMP-2/9 expression is highly context dependent across tumor types (e.g. pancreatic versus brain or prostate cancer), between stromal and malignant compartments, and over the course of therapy; constructs that assume uniformly “high” MMP-2/9 can be under-activated in low-expressing tumors, whereas broadly cleavable peptide linkers risk off-target activation in inflamed or remodeling normal tissues. These sources of heterogeneity lead to variable activation thresholds and increase the risk of premature, incomplete or misplaced transformation, particularly for single-stimulus systems; multi-stimulus designs can mitigate this at the cost of added synthetic and analytical complexity. Furthermore, the long-term biosafety of residual metallic or polymeric fragments remains insufficiently studied, and scaling production to GMP standards requires modular and reproducible chemistries. Overall, stimuli-responsive size and charge modulation presents a powerful strategy to reconcile systemic stability with deep tumor penetration, but future efforts must focus on tuning activation windows to clinically relevant conditions while simultaneously streamlining manufacturing and strengthening safety evaluation to enable reliable translation.

**Table 1:** Summary of size and charge strategies

System name	Mechanisms	Experimental animal model	Size (nm)/ζ-potential changes (mV)		Penetration model	Antitumor efficacy (tumor size/weight reduction)	Ref
MSN@SP-C9 (pH-shrinkable)	pH-triggered shrinkage of mesoporous silica carriers	4T1 tumor-bearing mice	~ 33 → ~ 17.84 nm		Tumor Sects. (24 h): vessel staining	Tumor inhibition rate 70.0%	[22]
TA/cystamine nanoassemblies	Dual pH/GSH-responsive disassembly of noncovalent assemblies	4T1 tumor-bearing mice	~ 122 → ~ 10 nm		3D 4T1 tumor spheroids: fluorescence detectable near spheroid center	Tumor inhibition rate ~ 89%	[23]
Au-Fe₂C nanocapsules (PFP@Au-Fe₂C-SRF)	Acid/thermal degradation of the shell releases small Au-Fe₂C cores and drives a ferroptosis cascade	Mouse xenografts	~ 320.2 → ~ 40.9 nm		Multicellular spheroids	—	[21]
(Te/QAm polycarbonate) @ HA	HAase-triggered shell removal leads to charge reversal (from negative to positive) with slight shrinkage	PANC-1 xenograft (3.6 mm thick)	—		Tumor Sects. (10 μm, confocal)	Tumor inhibition rate ~ 87%	[25]
Met@BF nanohybrid	Acid-activated charge reversal combined with ultrasound (US) piezocatalysis (BaTiO₃) to enhance chemodynamic therapy and antitumor immunity	Melanoma spheroids	− 16.6 → + 18.5 mV		Tumor spheroids: penetration distance ≈60 μm from periphery toward avascular core	Tumor inhibition rate ~ 87%	[27]
BBT-HASS@FPMPL NPs	pH-triggered charge reversal with HAase/GSH-induced collapse; releases the NIR-II photothermal dye	4T1 tumor-bearing mice	~ 299 → ~ 209 nm		4T1 spheroids (CLSM z-stack): fluorescence throughout spheroids	—	[29]
LPHF amphiphilic polymer (dual-transform)	At pH 6.5 the polymer precipitates to form a local depot, followed by regeneration of ≈33 nm cationic FA-targeted spheres	4T1 tumor-bearing mice		~ 18 → ~ 33 nm	Ex vivo tumor tissue penetration	Tumor inhibition rate 95.8%	[30]

Size values are taken directly from the cited studies and primarily represent hydrodynamic diameters measured by DLS under the indicated stimuli/time. Apparent 'shrinkage' typically arises from responsive shell contraction or disassembly into smaller building blocks/cores rather than compression of an inorganic core

These constraints argue for explicit integration of heterogeneity into design criteria. First, trigger thresholds should be tuned to clinically observed ranges, for example by combining mildly acidic pH (≈6.8–7.0) with a second cue such as elevated GSH, ROS or specific proteases, so that neither physiological tissues nor weakly altered regions alone are sufficient to activate the system. Logical AND-gated architectures, in which two or more abnormal cues must coexist to complete size shrinkage, charge reversal or ligand unmasking, can sharpen lesion specificity while remaining robust to local fluctuations. Conversely, redundant or OR-gated triggers (e.g. linkers cleavable by multiple MMPs and cathepsins, or dual pH/enzymatic responses) help maintain function across tumors with divergent expression profiles [33]. Second, coupling endogenous cues with externally applied energy sources (light, ultrasound, magnetic or electric fields) allows operators to override unfavorable local conditions and confine activation to image-guided target volumes [34]. Third, systematic mapping of pH, protease activity and redox gradients in patient-derived organoids and ex vivo tissues should be used to calibrate activation windows and to validate that transformation occurs in clinically relevant microdomains rather than only in idealized models. Overall, tumor heterogeneity does not negate the value of transformable systems, but it mandates multi-input logic, threshold tuning and model-informed design if stimuli-responsive behavior is to translate into reliable deep penetration in patients [35].

Across these acid-, redox- and enzyme-activated transformers, several trends emerge when considered together (Table 1). Simple pH/GSH shrinkers such as MSN@SP-C9 and TA/cystamine assemblies provide relatively straightforward chemistries and already improve penetration into collagenous matrices, but their depth of transport is typically limited to hundreds of micrometres and relies heavily on the presence of strongly acidic pockets. Protease-addressable ultra-shrinkers and DMMA-type charge-reversal systems introduce greater synthetic complexity but can achieve more dramatic size/charge switches and more durable retention in desmoplastic tumors, as illustrated by the multi-millimetre traversal of collagenase-armed CMSNs and the uniform penetration of HAase-responsive Te/QAm polycarbonates in thick xenografts. Dual-transform architectures such as BBT-HASS@FPMPL and the LPHF depot-regeneration strategy provide the most balanced profiles: they leverage initial shrinkage to lower interstitial resistance and then expose mildly cationic or ligand-decorated surfaces to secure uptake and pharmacodynamic gain, achieving up to ~ 96% tumor growth inhibition relative to the main control arms in aggressive murine tumor models, as reported in the original studies. However, these multi-trigger designs also sit at the high end of formulation complexity and CMC burden, underscoring the trade-off between depth of penetration, on-target retention and manufacturability [36]. Unless otherwise specified, tumor inhibition percentages and tumor-weight reductions cited in this review refer to improvements relative to each study’s primary control group (e.g. saline, free drug or non-transformable nanocarriers) as described in the source articles.

It is also important to interpret reported penetration distances in the context of the experimental model. Many platforms are first benchmarked in multicellular spheroids, where penetration on the order of 50–200 μm typically reflects diffusion from the periphery toward an avascular core with relatively uniform composition and short transport length scales [37]. By contrast, “millimeter-scale” penetration in rodent xenografts usually denotes coverage across the radius or thickness of a small murine tumor nodule, which spans only a few millimeters in size and has a distinct vascular and stromal architecture compared with human lesions [38]. These readouts are therefore best viewed as relative performance metrics within each model (e.g., shrinkers versus non-shrinkers, ligand-decorated versus undecorated carriers) rather than as direct predictors of absolute penetration depth in patients. In human solid tumors, centimeter-scale dimensions, higher interstitial fluid pressure, and more complex stromal organization mean that spheroid or mouse data can at most indicate the potential to improve local distribution under favorable conditions, and should be integrated with imaging and pharmacokinetic data when assessing translational promise [39].

### Stability and scalability

For transformable nanoformulations, stability should be assessed at both the formulation level and the biological/storage level. At the formulation level, key risks include colloidal instability, size and polydispersity index (PDI) drift, premature switching under non-target conditions, and unintended payload leakage during storage or circulation [40]. At the biological/storage level, serum stability and shelf-life are essential; practical measures such as optimizing PEG density and anchoring, reinforcing reversible crosslinks or coating integrity, and applying lyophilization or controlled freezing with appropriate cryo-/lyoprotectants can improve robustness when long-term storage is required. From a scalability perspective, platforms with relatively simple compositions and modular fabrication routes (e.g., nanoprecipitation or microfluidic mixing) are generally more amenable to scale-up/scale-out because critical process parameters can be systematically controlled to ensure reproducibility, including component ratios, concentration, flow-rate ratio, total flow, temperature, and solvent removal. In contrast, multi-component bioinspired constructs (e.g., membrane-coated particles and vesicle-derived systems) introduce additional challenges related to raw material variability, consistent functional presentation, sterilization compatibility, and quality control [41]. Therefore, incorporating manufacturability-oriented design early, minimizing unnecessary complexity, defining release specifications, and adopting continuous or semi-continuous processing strategies can improve translation readiness [42].

## Barrier overcoming systems

A growing set of platforms expands intratumoral distribution beyond passive enhanced permeability and retention (EPR) by coordinating vascular access, interstitial transport, and immune engagement, rather than relying on size alone. The tumor microenvironment combines poorly perfused and tortuous vessels, elevated IFP, a collagen-rich matrix, and immunosuppressive cells, which together distort drug gradients and create protected core regions that resist therapy. Contemporary barrier-overcoming platforms thus seek to-engineer one or more of these elements, thereby synchronizing perfusion, transport, and immune engagement with cytotoxic payload delivery. These mechanisms, see Table 2, converge on relieving transport bottlenecks across tumor compartments. Conceptually, effective designs now couple a short perfusion window with matrix softening or pressure relief, then layer cellular uptake and immune activation so that distribution and action are temporally aligned. Tumor vessels are structurally and functionally abnormal, with irregular branching, poor pericyte coverage and leaky junctions that jointly increase interstitial fluid pressure and impair convective transport. The concept of “vascular normalization” proposes that judicious, often low-dose anti-angiogenic modulation can transiently restore vessel integrity and perfusion, thereby enlarging the delivery window for drugs and nanocarriers [43]. This paradigm and its impact on drug delivery have been extensively reviewed elsewhere, including strategies based on VEGF/Ang2 blockade, PHD2 inhibition and metronomic chemotherapy regimens [44]. Here we focus on representative examples that directly document improved nanoparticle transport. As summarized in Fig. 1, barrier-overcoming platforms primarily operate at the vascular and ECM/IFP levels and usually represent the earliest step in the temporal sequence (priming before carrier transformation and payload activation). To enable rigorous cross-platform evaluation and to address reviewer concerns regarding quantitative comparisons, Table 2 now consolidates standardized transport/penetration metrics (e.g., penetration distance, spatial coverage/distribution surrogates, and tumor exposure-type readouts when reported) and explicitly states the comparator arm for each readout (e.g., PBS/vehicle, free drug, or non-transformable nanocarriers).

**Table 2:** Barrier-overcoming systems

System name	Mechanisms	Experimental animal model	Primary barrier targeted	Penetration model/key readouts in tumors	Antitumor efficacy (tumor size/weight reduction)	Ref
Nintedanib silicasome	Tyrosine-kinase inhibitor delivery; vessel pruning; collagen reduction; immune reprogramming	Orthotopic pancreatic cancer (mouse)	Abnormal tumor vasculature and stromal fibrosis	Tumor tissue (24 h): nintedanib quantified by HPLC (20.82 μg·g⁻^1^)	—	[45]
pre-GP1 “fibrous glue”	Bispecific peptide binding to NRP1 and PD-L1; in situ beta-sheet nanofiber assembly; vascular support	Preclinical renal carcinoma models	Vascular instability and perivascular immune exclusion	3D tumor spheroids: permeability factor increased by 6 times	Tumor inhibition rate 60.90%	[46]
LGK974 blood–brain barrier (BBB) modulation	Porcupine inhibition; temporary Wnt/beta-catenin blockade; restoration of caveolae-mediated transcytosis	Orthotopic CT2A and RCAS/tv-a glioma	Blood–brain barrier transcytosis brake (MFSD2A-dependent)	Tumor tissue: quantitative PTX (increased by 2.3–2.6 times)	—	[47]
ADM blockade	Inhibition of TAM-derived adrenomedullin; stabilization of endothelial adherens junctions	Tumor-bearing mice	Vascular hyperpermeability and junction disruption	—	—	[48]
Collagenase@CMSNs4.5 (DOX)	Collagen degradation on large-pore cationic mesoporous silica; enhanced doxorubicin delivery	Pancreatic tumor in vivo	Dense collagen-rich extracellular matrix	Tumor-slice depth profiling; detectable penetration depth: 1200 μm	Tumor inhibition rate 86%	[49]
HA-DOX@GNPs-Met@HFn	MMP-2-triggered downsizing and release of Met@HFn; AMPK activation; TGF-β downregulation; CAF depletion	Tumor-bearing mice	Collagen/CAF stroma and solid stress	—	Tumor inhibition rate ~ 87%	[50]
ENP5 fusiform CaP	High-curvature geometry (apical mean curvature 1,004.8 µm⁻^1^) to reduce viscous drag	Huh-7 hepatocellular carcinoma	Pressure-limited extravasation and matrix tortuosity	Matrix transport assay; penetration enhancement factor increased by 12.2 times	—	[51]
RPC@M nanozyme	Photocatalytic water splitting lowers interstitial fluid pressure; resveratrol reduces collagen and solid stress	Tumor-bearing mice	Interstitial fluid pressure and solid stress (TIFP and TISP)	CLSM spheroid penetration; penetration depth: 80 μm	—	[52, 53]

### Vascular normalization

**Fig. 3:**
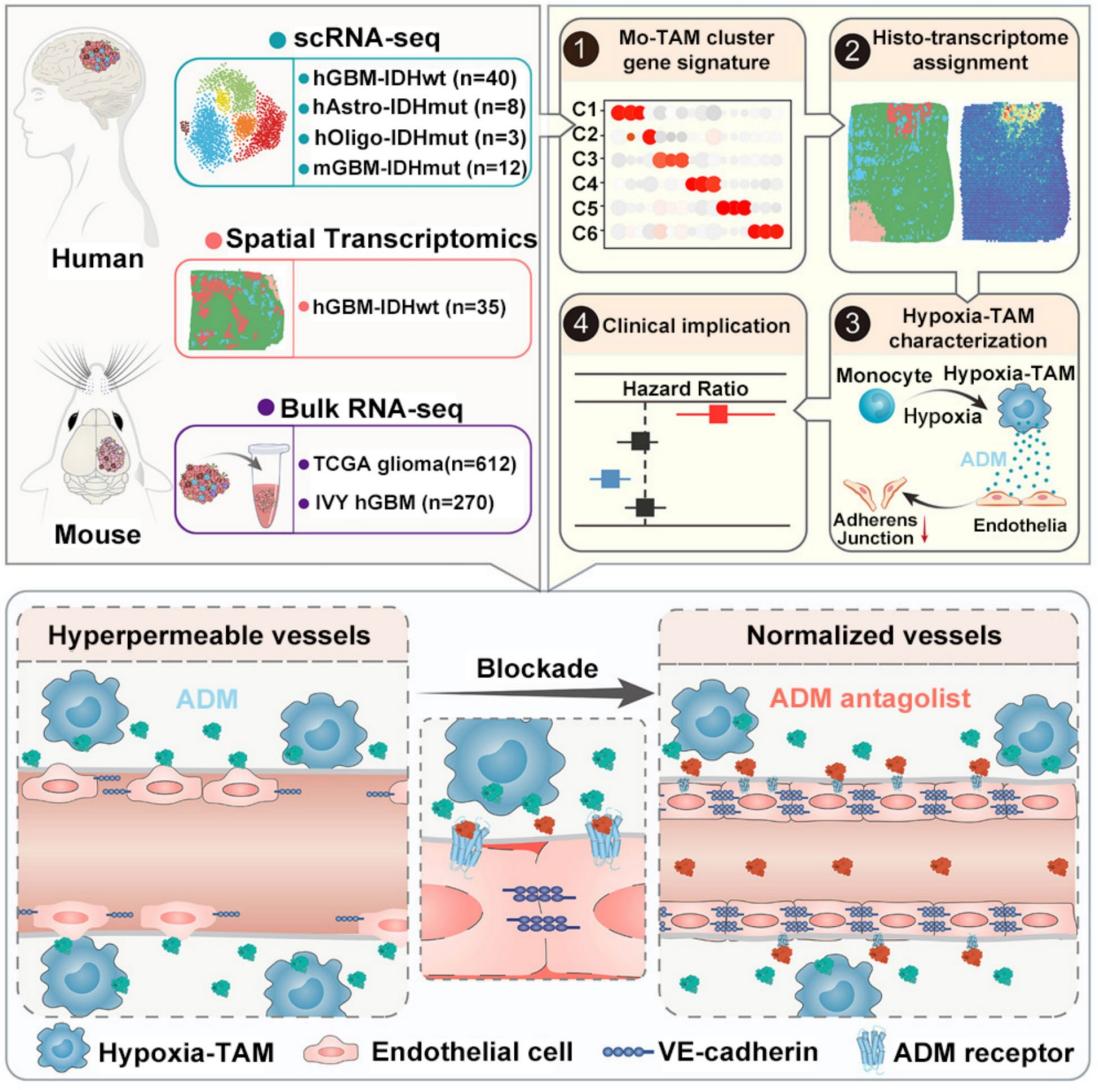
Hypoxia-associated tumor-associated macrophage-derived adrenomedullin (ADM) destabilizes endothelial adherens junctions via the calcitonin receptor-like receptor (CRLR), leading to vascular hyperpermeability. Blocking ADM restores vessel integrity, enhances intratumoral drug delivery, and improves antitumor efficacy. *Adapted from Wang, W., *et al*., Cancer Cell 42 (2024) 815–832, * 10.1016/j.ccell.2024.03.013*.* Licensed under CC BY 4.0 (https://creativecommons.org/licenses/by/4.0/). Changes were made [48]

Among the barrier-focused approaches, vascular normalization is one of the most well established. Moderation rather than maximal anti-angiogenesis yields the most useful delivery window (Fig. 3). By transiently restoring endothelial integrity and lowering shear stress, controlled release of vasoactive cues such as nitric oxide(NO) or tyrosine kinase inhibitors (TKIs) can increase perfusion and nanoparticle flux [52, 53]. Clinically, however, the “normalization window” is difficult to time because its onset and duration vary across tumor types, baseline vascular abnormalities, and the class/dose/schedule of vascular-modulating agents; therefore, longitudinal monitoring with functional perfusion/permeability imaging and circulating angiogenesis-related biomarkers is often explored to infer when the window is open and guide combination scheduling. Transient restoration of endothelial integrity and flow reduces hydrodynamic resistance and increases nanoparticle flux. A silicasome that delivers the multi-target TKI nintedanib increases intratumoral drug exposure, prunes aberrant vessels, reduces collagen, elevates the CD8⁺/FoxP3⁺ ratio, and enables synergy with anti-PD-1 in a robust orthotopic pancreatic cancer model [45], with efficacy evaluated against (i) free nintedanib (systemic/oral dosing in the source study) and (ii) checkpoint-inhibitor monotherapy arms rather than an unspecified “control.” These data exemplify the idea that moderation, not maximal anti-angiogenesis, yields the most useful delivery window, since excessive pruning compromises perfusion despite reducing IFP. Wu et al. exploited a bispecific precursor peptide (pre‑GP1) that binds neuropilin‑1 and PD‑L1, self‑assembling in situ into *β*‑sheet “fibrous glue.” In advanced renal carcinoma models, the key outcomes are improved vascular support and deeper intratumoral distribution, with penetration/efficacy reported relative to antibody-only comparators (e.g., PD-L1 antibody) and to the clinical combination regimen used as a benchmark in that work (Table 2), increased perivascular CD8⁺ T cells 11.3 fold and outperformed the bevacizumab plus atezolizumab standard in growth and metastasis control in preclinical renal carcinoma [46]. At the blood–brain barrier, Xie et al*.* transiently blocked endothelial Wnt/β-catenin with the porcupine inhibitor LGK974 (2.5 mg kg⁻^1^ × 4 days) in orthotopic CT2A and RCAS/tv-a glioma, downregulating major facilitatorsuperfamily domain-containing protein 2 A (MFSD2A) and unleashing caveolae-mediated transcytosis across the blood–brain barrier. The reported increase in intratumoral TMZ and paclitaxel levels is presented in Table 2 explicitly as “LGK974 + drug” versus “drug alone with standard systemic dosing (no. LGK974)” to clarify the control condition [47]. Together, these examples illustrate that normalization can be achieved by vascular support, junctional stabilization or selective pathway interruption, but all require careful control of dose and timing to preserve perfusion while lowering transport resistance. Beyond these specific formulations, multiple studies have shown that partial vascular pruning and pericyte reinforcement, achieved with diverse TKIs, antibodies, and genetic modulators, can similarly normalize perfusion and enhance nanomedicine delivery across a range of tumor models [54, 55].

### ECM and IFP remodeling

A broad range of pharmacological, enzymatic, and physical interventions aim to decompress vessels, relax stromal scaffolds, and lower interstitial resistance [56]. In this section, we focus on two complementary routes: (i) barrier-relief interventions that directly reduce ECM/solid stress and (ii) carrier designs (shape/size/stiffness) that exploit these changes to improve transport through remodeled matrices.

In solid tumors, IFP is markedly elevated (typically 10–40 mmHg), due to leaky vasculature and dysfunctional lymphatic drainage. This reduces the interstitial pressure gradient that drives convection, creating a hydrodynamic barrier to nanoparticle extravasation and tissue diffusion [56]. Collagenase-armed CMSNs traverse millimeter-scale collagenous stroma in small pancreatic xenografts (penetration ≥ 1.2 mm across the stromal compartment) and increase doxorubicin delivery [49], with penetration and antitumor readouts reported versus both PBS/vehicle controls and non-collagenase nanoparticle comparators in the source study (Table 2). Hybrid constructs that pair MMP-triggered downsizing with CAF depletion (HA-DOX@GNPs-Met@HFn) relieve solid stress while sustaining transport gains [50], and the spheroid penetration depth comparison is reported in Table 2 with clear comparator groups (free DOX/HA-DOX vs HA-DOX@GNPs-Met@HFn, with/without MMP-2 pretreatment as described). Geometry engineering via high-curvature fusiform CaP (ENP5) lowers hydrodynamic drag, enabling long-range matrix traversal (over ~ 1.5 mm across the perivascular matrix in Huh-7 xenografts) enabling long-range matrix traversal and potent antitumor activity, with extravasation/penetration metrics explicitly benchmarked against spherical CaP (and the relevant vehicle/free-drug controls used in that report) as summarized in Table 2 [51]. Dendritic polymer-based nanomedicines that phenotypically reprogram cancer-associated fibroblasts and decompress tumor vessels provide a complementary stromal-normalizing route, lowering solid stress, increasing perfusion and CD8⁺ T-cell infiltration, and thereby improving the efficacy of immune checkpoint blockade in desmoplastic lung tumors, where the therapeutic benefit is interpreted relative to both “checkpoint blockade only” and vehicle arms (rather than an implicit control) [57]. Operationally, most ECM-modulating approaches fall into enzymatic/catalytic loosening versus pressure-decompression regimens: (i) enzymatic/catalytic loosening of stromal scaffolds and (ii) physics-informed particle design that minimizes viscous drag and tortuosity losses. Enzymatic loosening has also been combined with energy-based therapy to alleviate stromal resistance. Collagenase-loaded hydrogenated TiO₂ nanoparticles softened collagen-rich tumor stroma, improved ultrasound imaging guidance, and augmented sonodynamic therapy by enabling deeper intratumoral penetration and drug action with therapeutic enhancement reported versus non-collagenase nanoparticle [58]. Mechanistic and computational analyses that couple cell traction, matrix degradation, and transport further support ECM remodeling as a route to reduce solid stress and increase effective diffusivity, providing a quantitative rationale for targeting collagen and hyaluronan to relieve interstitial flow barriers [59]. In the first category, agents such as hyaluronidase, PEGylated recombinant hyaluronidase (PEGPH20), losartan and TGF-β or LOX inhibitors have been shown to deplete hyaluronan, relax collagen networks and decompress vessels, thereby improving nanomedicine accumulation in desmoplastic tumors. In the second, shape-, size- and stiffness-engineered nanocarriers exploit reduced effective viscosity and altered steric interactions to traverse dense ECM, as demonstrated for high-aspect-ratio rods, deformable vesicles and curvature-engineered inorganic particles across multiple tumor models [60].

Despite these advances, each modality carries intrinsic limitations. Enzyme-based ECM depletion may risk off-target proteolysis or compensatory fibrosis; vascular normalization is transient and dose-sensitive; and spatial heterogeneity in TME composition complicates universal trigger design. Future work should integrate patient-specific barrier mapping with spatiotemporally controlled interventions and combination regimens that merge barrier removal with active targeting and immune activation, thereby converting microenvironment-informed design into durable therapeutic gain.

## Cell-based and bioinspired delivery systems

Intratumoral transport is restricted by abnormal vasculature, elevated IFP, and a dense ECM. Cell-based and biomimetic vectors leverage innate homing, transendothelial migration, chemotaxis, and immune evasion to improve accumulation, penetration, and on-site release. Table 3 lists cell-based and bioinspired vectors such as membrane-coated carriers, EVs, micromotors, neutrophil shuttles, CPP-assisted systems, alongside primary barriers targeted and intratumoral readouts. In the common taxonomy of Fig. 1, cell-based and bioinspired. vectors are characterized by biological activation and homing, with penetration spanning vascular, interstitial and cellular levels and often contributing to late-stage immune and microenvironmental remodeling. To enable cross-platform evaluation and to address reviewer concerns on qualitative-only summaries, we consolidate available *standardized transport metrics* (e.g., penetration distance in 3D models, BBB crossing efficiency, diffusion coefficients, intratumoral deposition fold-change) and explicitly state the comparator context (vs. PBS/saline, vs. free drug, or vs. non-motile/non-biomimetic controls) in Table 3.

**Table 3:** Cell-based and bioinspired delivery systems

System name	Mechanisms	Experimental animal model	Primary barrier targeted	Penetration model/key readouts in tumors	Antitumor efficacy (tumor size/weight reduction)	Ref
PLGA-STM-TAT@CCM-YSA	Cancer-cell-membrane cloaking for homologous adhesion and immune camouflage; EphA2 targeting (YSA); METTL3 inhibitor delivery with TAT; suppression of c-MYC/BRD4 and PD-L1; synergy with anti-PD-1	Tumor-bearing mice (EphA2-high lesions)	Immune recognition and intralesional retention	—	—	[61]
DC-derived extracellular vesicles loaded with VEGF-A siRNA and doxorubicin	Concurrent anti-angiogenic siRNA and chemotherapy; conservation of EV markers (CD9/CD63/CD81); vascular normalization after intranasal dosing	Orthotopic glioma (rodent)	Abnormal vasculature and perfusion	In vivo fluorescence: VEGF-A 323.65 → 77.50 pg/mL	—	[62]
PLT@Au@Urease platelet micromotors	Urease-driven self-propulsion; enhanced cellular uptake; gold radiosensitizer payload	4T1 tumor-bearing mice	Endothelial and stromal hindrance; limited uptake under static conditions	Motility/diffusion: D 0.03 → 2.50 μm^2^/s; radiosensitization SER: 1.89	—	[63]
Neutrophil “Trojanbot” carrying catalase-powered nanobots (CatNbots)	Neutrophil chemotaxis for BBB crossing; H₂O₂-driven self-propulsion of released CatNbots in tumor	GL261 orthotopic glioma	Blood–brain barrier and interstitial tortuosity	In vivo tumor fluorescence accumulation 5.1 ×	—	[64]
PMN/T7/TMZ cell nanocarriers	Neutrophils adsorbed with transferrin-targeted T7 micelles; NET-triggered payload release	GL261-luc orthotopic glioblastoma	Blood–brain barrier and tumor homing	IVIS bioluminescence: total flux; NR	—	[65]
PEG-CPP33-decorated ZIF-90 MOFs (oridonin (ORI) + survivin siRNA)	CPP-enhanced membrane engagement and endocytic entry; acid-triggered release; improved lysosomal escape; chemo-gene synergy	Tumor cell lines and mouse models	Cellular entry and endosomal/lysosomal escape	A549 tumor model: survivin mRNA downregulation 69.34 ± 1.06%; NR	Tumor inhibition rate: 61.04%	[66]
Tat-A86 elastin-like polypeptide nanoparticles (with AP1; miRNA cargo)	Stable peptide-nucleic-acid complexation; CPP-assisted penetration in 3D tissue	LLC tumor models and spheroids	Cellular entry and 3D tissue penetration	LLC spheroids: fluorescence distribution; NR	Tumor inhibition rate: ~ 38%	[67]
RMMR1-decorated PEG-PLA micelles (etoposide + quercetin), intranasal	CPP raises uptake via nose-to-brain transport; quercetin inhibits P-gp efflux to increase brain exposure	Rats and related models	Nose-to-brain delivery and efflux (P-gp)	Intranasal dosing: brain exposure; NR	—	[68]
ΔppGpp-attenuated Salmonella typhimurium expressing hyaluronidase	In situ secretion of Staphylococcus hyaluronidase to degrade hyaluronan; reduction of interstitial fluid pressure; potentiation of chemotherapy	4T1 breast cancer and ASPC-1 pancreatic models in mice	Hyaluronan-rich extracellular matrix and elevated interstitial fluid pressure	HA depletion markers & tumor IFP reduction; NR	—	[69]
RPC@M nanozyme (tumor-cell-membrane coated resveratrol@polydopamine cobalt phytate)	Photocatalytic water splitting under six hundred sixty nanometer irradiation lowers interstitial fluid pressure; resveratrol suppresses collagen deposition to reduce solid stress; membrane camouflage supports homologous adhesion	Tumor-bearing mice	Interstitial fluid pressure and solid stress; stromal collagen	Tumor water proportion (IFP proxy); collagen-related readouts; NR	—	[53]

NR: *Not reported*. The original reference did not explicitly report a quantitative value/metric for this item in the text

### Microenvironment priming systems

These systems prepare the tumor microenvironment to restore perfusion, lower IFP, and ease diffusion. Cancer-cell membrane cloaks provide homologous adhesion and immune camouflage, which concentrates delivery inside EphA2-high lesions and reshapes oncogenic and immune programs. A representative construct (PLGA-STM-TAT@CCM-YSA) illustrates this pattern, preferential deposition with transcriptional rewiring and anti-PD-1 synergy [61]. Membrane-disrupting polymers (e.g., amphiphiles inserting into lipid bilayers) can weaken cell–cell junctions and induce NanoEL-like permeability, as quantified by TEER decline and Transwell leakage, thereby facilitating deeper transport and subsequent therapy [70]. In vivo, the combined administration inhibited tumor growth most significantly *compared with anti-PD1 antibody alone* (and vs. PBS control), clarifying the control context for the reported synergy. Extracellular vesicles (EVs) offer a complementary route in which vascular normalization and immune recruitment are encoded by vesicle cargo and surface markers. Dendritic-cell macrovesicles loaded with VEGF-A siRNA and doxorubicin conserved CD9/CD63/CD81, enhanced uptake and normalized the vascular bed, curbing vasculogenic mimicry and drawing perivascular T cells in orthotopic glioma [62]. Consistent with reviewer requests, the comparative framing is explicit: the authors conclude this EV system is more potent than bevacizumab (BV) alone, i.e., the referenced anti-VEGF mAb control. Photosynthetic Chlorella loaded with indocyanine green generates oxygen under 660 nm irradiation and splits interstitial water, which lowers interstitial fluid pressure, improves perfusion, and deepens doxorubicin penetration in vivo. Under 808 nm irradiation the same platform adds photothermal and photodynamic effects, and together with starvation effects nearly eradicates tumors in mice while maintaining a favorable safety profile. This catalytic priming complements membrane coated and extracellular vesicle based approaches by directly modulating tumor hydraulics before cellular uptake and release. Engineered bacteria that secrete hyaluronidase degrade hyaluronan in situ, reduce interstitial fluid pressure, and potentiate chemotherapy in breast and pancreatic tumor models with survival extension [69]. In these bacterial-ECM priming studies, tumor growth suppression is reported *relative to PBS* and to drug-only arms (e.g., doxorubicin or gemcitabine alone), with the strongest effects in combined HAase-bacteria + chemotherapy groups. A tumor cell membrane coated resveratrol and polydopamine cobalt phytate nanozyme lowers fluid pressure by photocatalytic water splitting under 660 nm and reduces solid stress by suppressing collagen deposition, which together increases deep penetration and enables strong in vivo ablation without systemic toxicity [53]. Mechanistically, TIP reduction was supported by decreased tumor water proportion (TIFP proxy) and reduced Col1a1 expression vs control, providing a quantitative “barrier-relief” readout even when penetration distance is not reported.

Despite these impressive preclinical readouts, exosomes, cell-membrane-coated nanoparticles, macrophage-mediated carriers and engineered bacteria remain far from manufacturing and regulatory maturity [71]. Exosome and membrane preparations often exhibit batch-to-batch variability in vesicle size, lipid and protein composition and functional marker expression, and their activity is highly sensitive to isolation methods, storage conditions and freeze thaw cycles. Preserving the orientation and function of membrane proteins while removing residual intracellular content, nucleic acids, endotoxins and replication-competent contaminants is technically demanding and requires stringent sterility and pyrogen testing. Scaling up production under GMP conditions is further complicated by the need for large, well-characterized cell banks or microbial strains and robust in-process controls, and by the multi-origin nature of many hybrid constructs, which blurs the boundary between biologics, devices and combination products in current regulatory frameworks [72]. These issues do not negate the translational promise of bioinspired vectors, but they underscore that advances in process analytics, standardized characterization panels and regulatory guidance must progress in parallel with materials design.

### Barrier navigation systems

**Fig. 4:**
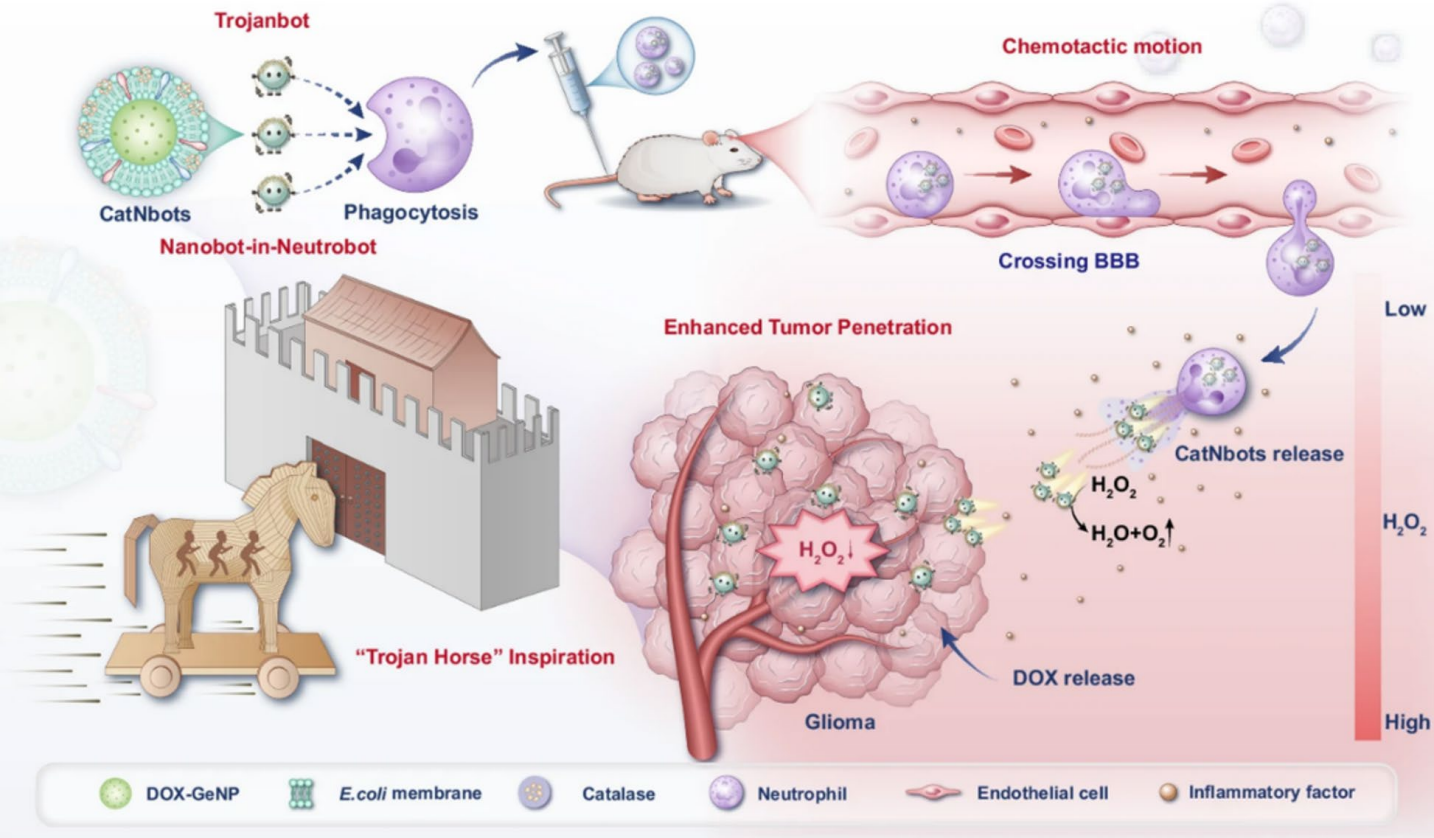
Neutrophil “Trojanbot” ferries catalase-powered nanobots across the blood-brain barrier via chemotaxis, and upon release in the tumor microenvironment the nanobots self-propel along hydrogen peroxide gradients to achieve deep tumor penetration and enhanced doxorubicin delivery in glioblastoma. Reproduced from Gao, Y., et al., Nature Communications 16 (2025) 5263, 10.1038/s41467-025-60422-z. © The Author(s) 2025. Licensed under CC BY-NC-ND 4.0 (https://creativecommons.org/licenses/by-nc-nd/4.0/). No changes were made [64]

These systems actively navigate endothelium, stroma, and the blood–brain barrier (BBB). Platelet micromotors (PLT@Au@Urease) carried about 29 pg of Au radiosensitizer per platelet and showed zeta potential near − 17.5 mV. In physiological urea they transitioned from Brownian diffusion (D ~ 0.03 μm^2^ s⁻^1^) to about 2.5 μm^2^ s⁻^1^, doubled cancer-cell uptake versus static PLT@Au, and in 4T1 tumors a single intratumoral dose plus 4 Gy *γ*-irradiation raised the sensitization enhancement rate to 1.89 (vs. 1.08 for passive PLT@Au), producing the largest 14-day tumor weight reduction without extra toxicity [63]. For BBB traversal and deep brain delivery, neutrophil “Trojanbots” encapsulated catalase-powered gelatin nanobots (CatNbots). The assemblies measured about 329 nm (*ζ* ~ − 35.6 mV), crossed an in vitro BBB with roughly 50% efficiency, and once released self-propelled about 416 μm through GL261 spheroids. To clarify comparator context, the source reports BBB penetration of ~ 50% (≈10 × vs GeNPs) and spheroid penetration distance of ~ 416 μm (≈2.8 × vs GeNPs). In orthotopic glioma they increased drug deposition nearly fivefold and extended median survival (median survival 38 days; vs PBS 18 days, EMV@GeNP 25 days, CatNbots 26 days, and NE@EDGNs 34 days) to 45 days without organ toxicity [64] (Fig. 4). Similarly, PMN/T7/TMZ “cell nanocarriers” were created by adsorbing transferrin-targeted T7 cholesterol micelles (about 153 nm, *ζ* ~ + 3 mV) onto 1 × 10⁷ murine neutrophils. After intravenous infusion in GL261-luc orthotopic glioblastoma, the carriers crossed the BBB, homed to tumor foci, and used NET formation to trigger payload release. Bioluminescence decreased most at days 7 and 14, outperforming free TMZ, T7/TMZ micelles, or neutrophils alone, with no added toxicity [65]. Collectively, such “neutrophil shuttles” exemplify a broader class of neutrophil-based delivery platforms that harness innate chemotaxis, transendothelial migration, and neutrophil extracellular trap formation for targeted nano-drug transport across vascular and stromal barriers, as comprehensively reviewed elsewhere. [73] Taken together, the barrier-navigation systems in Sect. "Barrier navigation systems" occupy complementary niches (Table 3). Enzyme-powered platelet micromotors mainly enhance local convection and cellular uptake after intratumoral administration, providing strong radiosensitization with limited systemic exposure but also constrained reach beyond the injection field. Neutrophil “Trojanbots” and PMN/T7/TMZ carriers, by contrast, exploit active homing and BBB transmigration to distribute payloads widely within orthotopic glioma; they offer superior brain delivery and survival benefit, at the price of more demanding cell sourcing, processing and quality control. Across platforms, the most directly comparable transport endpoints include diffusion/propulsion (D), 3D penetration distance (μm), BBB crossing efficiency (%), and intratumoral deposition fold-change (×), which we tabulate to support a more rigorous cross-study comparison despite differences in models and imaging pipelines (Table 3). Translational limitations of barrier-navigation systems should be parsed into biological versus manufacturing/CMC constraints, particularly for “living” carriers such as neutrophil shuttles and Trojanbots. Biologically, performance and safety may be impacted by unintended immune activation and inflammatory toxicities, including premature neutrophil activation and NETosis, off-target tissue injury, altered trafficking/biodistribution, and inflammation-dependent variability across disease states and patients. From a manufacturing perspective, the regulatory pathway more closely resembles cell-based combination products than conventional liposomes: ex vivo neutrophil isolation and loading require stringent controls for source and starting-material variability, identity/purity and functional-state assays, loading consistency, viability, sterility/endotoxin testing, and robust comparability across batches and process changes during scale-up and storage [74].

### CPP assisted bioinspired systems

Cell-penetrating peptides (CPP) act as bioinspired modules that strengthen membrane engagement, endocytic entry, and endosomal escape, and can also support adsorptive-mediated transcytosis at endothelium. Across materials classes (MOFs, elastin-like polypeptides, polymeric micelles, mesoporous silica), CPPs consistently shift the rate-limiting step from membrane access to intracellular trafficking, with brain-directed designs further exploiting adsorptive-mediated transcytosis (AMT) and efflux modulation. PEG-CPP33-decorated zeolitic imidazolate framework-90 (ZIF-90) MOFs co-deliver small molecules and siRNA with acid-triggered release and improved lysosomal escape to produce chemo-gene synergy [66]. Tat-A86 elastin-like polypeptide nanoparticles presented AP1 together with a CPP to form stable miRNA complexes, increasing 3D spheroid penetration and suppressing Lewis lung carcinoma (LLC) growth at equivalent doses [67]. For CNS delivery, CPP-decorated micelles (e.g., RMMR1 PEG-PLA) and TAT-functionalized silica demonstrate nose-to-brain access and BBB penetration when paired with efflux inhibition or AMT [68, 75]. Where quantitative “penetration distance” or “area coverage” metrics are reported in the primary studies, we list them in Table 3; otherwise, we keep the description qualitative but maintain explicit control comparators when efficacy statements are made.

From a safety perspective, CPP-containing and other strongly cationic nanoparticles increase the risk of dose-dependent cytotoxicity, hemolysis, complement activation and non-specific adsorption to plasma proteins and vascular endothelium, which can trigger undesired uptake in the blood circulation, rapid RES sequestration and increased exposure of healthy tissues [76]. Stimuli-responsive motifs that generate reactive oxygen species, heat or membrane-lytic fragments can further narrow the therapeutic window if activation is not tightly restricted to the target volume [77]. In addition, peptide synthesis and conjugation impose sequence-specific quality control requirements, while protease susceptibility, serum binding and binding-site barriers can restrict tissue spread and distort biodistribution profiles. These risks must be countered with tumor-activated masking of cationic domains, rigorous control of CPP density and orientation, careful tuning of trigger thresholds and incorporation of pH- or enzyme-responsive unshielding and escape modules. Despite these hurdles, the strategic upside is compelling: cell-based and biomimetic systems can bypass endothelium and localize actively, and when augmented with CPP modules they achieve efficient cellular entry and endosomal escape to enable deep and uniform penetration. The path to translation is to advance artificial cell mimetics, standardized membrane reconstitution, modular EV polymer hybrids and activatable, clinically scalable CPP chemistry together with robust release testing, stability standards and in vivo immunogenicity maps [76].

## Ligand and transcytosis mediated penetration

**Fig. 5:**
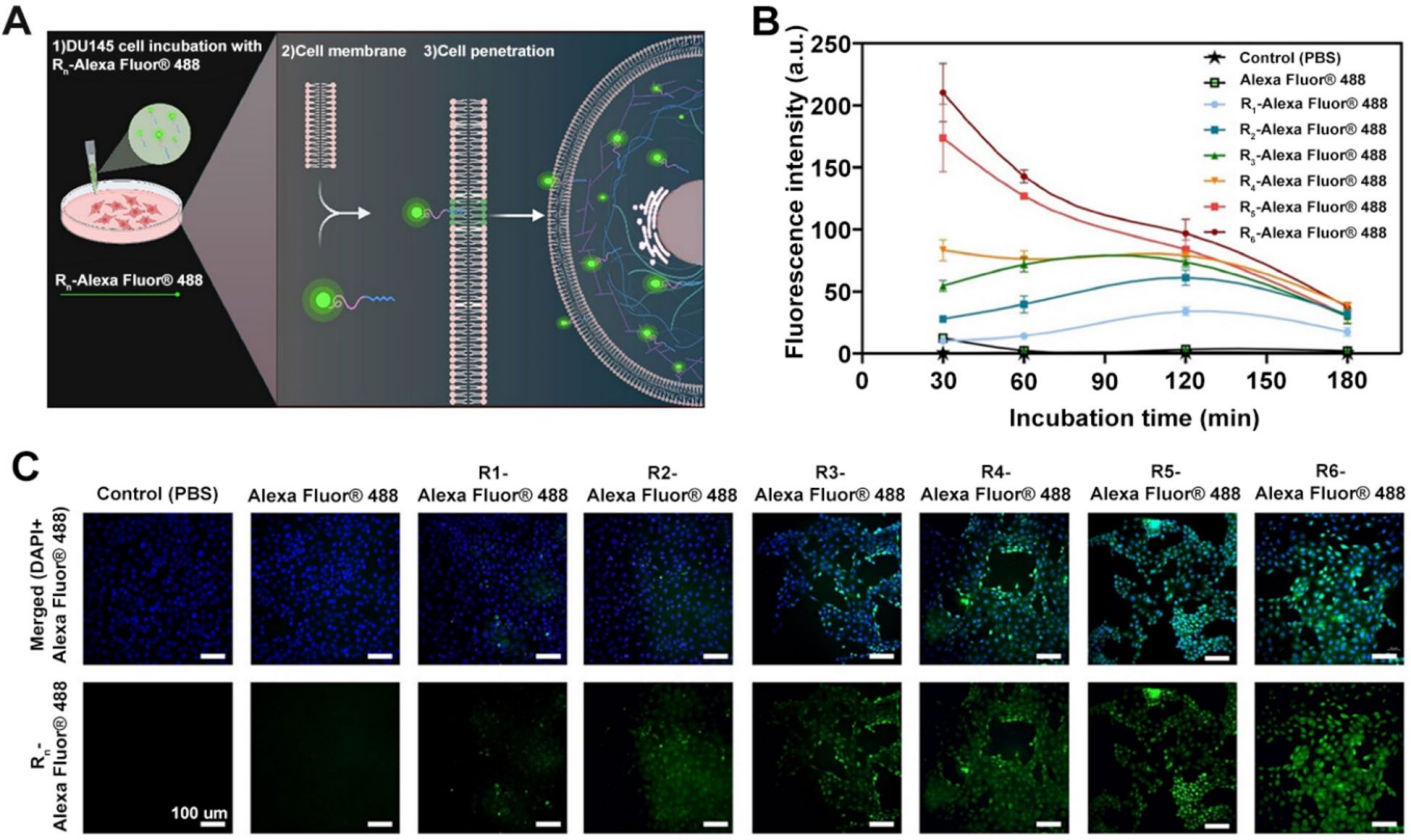
Schematic of ligand- and enzyme-triggered penetration. The R6-AANCK-CBT precursor is cleaved by legumain, exposing reactive groups that drive intracellular assembly into nanostructures and enhance transport across tissue barriers and intracellular delivery. *Adapted with permission from Ghaemi, B.; Tanwar, S.; Singh, A.; *et al*., ACS Applied Materials & Interfaces 16 (2024) 11,159–11,171, * 10.1021/acsami.3c14908*.* © 2024 American Chemical Society. Permission under RightsLink License No. 6123170315940. Changes were made. [80]

A key limitation of nanoparticle therapy is heterogeneous tumor deposition driven by weak recognition/internalization and limited traversal through dense ECM [78]. The EPR effect alone is often inadequate. Ligand-functionalized platforms add active binding, trans-barrier transport, and facilitated uptake to improve selectivity and distribution [79] (Fig. 5). Ligand- and transcytosis-mediated systems occupy the “receptor-guided” branch in Fig. 1, where trigger-gated ligand exposure and CendR-mediated transcytosis govern vascular crossing and interstitial spread. To enable cross-platform comparison. Table 4 consolidates, where available, quantitative penetration/distribution proxies (e.g., spheroid-core access, tumor growth inhibition, tumor weight/volume relative to a clearly defined comparator, metastatic burden) and explicitly specifies whether the comparator is PBS/saline, free drug, or non-targeted carriers [74].

**Table 4:** Ligand & transcytosis systems

System name	Mechanisms	Experimental animal model	Primary barrier targeted	Penetration model/key readouts in tumors	Antitumor efficacy (tumor size/weight reduction)	Ref
R6-AANCK-CBT (legumain-triggered intracellular self-assembly)	Legumain cleavage exposes reactive groups and intracellular CBT condensation forms nanostructures; increased intracellular retention and support for tissue transport after extravasation	DU145 and LNCaP prostate cancer cells; mouse acute-dose profiling	Protease-gated entry and perivascular to interstitial transport	Cell uptake/assembly; CP50 (R6) = 2.1 μM	—	[80]
iPR (iRGD-BRD4 PROTAC conjugate; glutathione-cleavable)	iRGD targets α_v_β_3_ integrins and, after proteolysis, exposes a CendR motif that binds NRP1 for transcytosis; a glutathione-responsive linker releases the PROTAC	MDA-MB-231; patient-derived organoids; tumor-bearing mice	Endothelial transcytosis and perivascular to interstitial transport	DU145 cell penetration	—	[81]
F3-TRAIL (nucleolin-targeted trimeric fusion)	F3 peptide targets surface nucleolin; multivalent presentation enhances death-receptor clustering and tumor penetration	Multiple tumor lines; mouse xenografts	Perivascular penetration and cellular uptake via nucleolin	Internalization kinetics + mechanistic modeling; NR	Tumor inhibition rate: 81.5%	[82]
cRGD-modified pH and ROS dual-responsive docetaxel nanoparticles	α_v_β_3_-targeted uptake combined with acetal and boronate cleavage enables deep penetration and on-site release	4T1 spheroids and mouse model	Cellular entry, endosomal or lysosomal processing, intratumoral penetration	4T1 spheroids; IC50: 15.57 ng/mL	—	[83]
T22-PE24 (CXCR4-targeted self-assembling nanotoxin)	T22 cell-penetrating ligand drives selective internalization in CXCR4-positive cells; PE24 toxin payload; induction of caspase-3 and GSDME-dependent pyroptosis with immune remodeling	Hepatocellular carcinoma models; immunocompetent and immunodeficient mice	Receptor-mediated cellular entry and intratumoral spread	3D 4T1 tumor spheroids (CLSM); NR	Tumor inhibition rate: 35%	[84]
T22-PE24 (independent or extended report)	Same mechanism as above	As reported	As reported	Tumor section IHC/IF; NR	—	[85]
cp-PCC (PCC16; CPP-induced chimera that degrades DHHC3)	Cell-penetrating chimera recruits CRBN to degrade palmitoyltransferase DHHC3, which destabilizes PD-L1	C33A cells; immune-checkpoint-blockade-resistant 4T1 xenografts	CPP-enabled tumor and cellular entry; functional penetration via PD-L1 pathway modulation	—	Tumor inhibition rate: 90.8%	[86]
Protease-activatable PL3 (uPA-dependent CendR exposure)	uPA cleavage unmasks the CendR motif while retaining tenascin-C binding; discovered with T7 phage and high-throughput sequencing	Orthotopic models reported in the literature	Protease-gated NRP1 transport with reduced off-target accumulation	—	—	[87]
IFN-γ mRNA@βGlus-LNPs	Early and loose extracellular matrix favors small particles through size-dominated transport; late and dense stroma favors larger multivalent particles through strong α_v_β_3_ engagement; size and ligand density should be tuned to stage	Staged orthotopic models	Endothelial crossing and stromal traversal adapted to tumor stage	Tumor section IHC; NR	Tumor inhibition rate: 59.2%	[88]

NR: *Not reported*. The original reference did not explicitly report a quantitative value/metric for this item in the text

### Tumor penetrating ligand systems

Across integrin-, nucleolin-, and CXCR4-addressed designs, two shared effects dominate: (i) increased endothelial access and perivascular-to-interstitial spread (often via CendR/NRP1 pathways), and (ii) reinforced intracellular processing (e.g., PROTAC delivery or toxin activation) that converts deeper distribution into pharmacodynamic gain. iRGD conjugation to a BRD4 PROTAC via a GSH-responsive disulfide carbonate linker (iPR) improves solubility, enables α_v_β_3_/NRP1-dependent uptake and CendR permeation, and strengthens BRD4 degradation and antitumor effects versus the parent small molecule [81]. In an MDA-MB-231 xenograft model, PRO achieved a TGI of 36.3% (vs PBS), whereas iPR reached 62.3% (5 μM) and 81.5% (8 μM) after 21 days (vs PBS), supporting a quantitatively stronger in vivo benefit than the parent PROTAC at matched/near-matched dosing. Fusing the nucleolin targeted F3 peptide to trimeric TRAIL creates a multivalent construct that improves tumor penetration and strengthens death receptor signaling, lowering IC50 values by about ten to 40- fold across several tumor lines and reducing tumor weight in vivo versus TRAIL alone [82]. Cyclic RGD-modified, pH/ROS dual-responsive docetaxel nanoparticles align targeting with stimuli-triggered disassembly to enhance intratumoral distribution and tumor control in 4T1 models [83]. Notably, in 4T1 tumor-bearing mice, DTX/RGD NPs yielded 0/6 tumors exceeding 600 mm^3^ at day 20 (vs 6/6 saline, 6/6 blank NPs, 4/6 free DTX, 3/6 DTX/PLGA NPs), and no metastatic clots were observed in lung after DTX/RGD NP treatment (vs abundant nodules in saline/blank groups), providing explicit comparator-anchored distribution/efficacy readouts beyond qualitative imaging. Beyond integrins, a CXCR4-targeted self-assembling nanotoxin (T22-PE24) selectively internalizes into CXCR4-positive hepatocellular carcinoma to trigger caspase-3/GSDME-dependent pyroptosis with immune remodeling [84, 85]. Likewise, tumor-penetrating peptides (e.g., iRGD/CendR pathway) have been broadly used as penetration-promoting modules in peptide-drug conjugates and nanocarriers; when combined with cleavable linkers (e.g., reducible/disulfide or enzyme-responsive motifs), they can improve intratumoral dispersion while helping limit off-target exposure [89].

### CendR-mediated tissue penetration systems

CendR logic generalizes ligand action from static binding to trigger-gated transcytosis and tissue flow. A CPP-induced chimera (cpPCC) recruits CRBN to degrade DHHC3 and indirectly destabilize PD-L1; the lead PCC16 achieved DC50 ~ 0.103 μM in C33A cells and reduced tumor weight to 10.1% of the PBS-treated control in ICB-resistant 4T1 xenografts, outperforming BMS-8 (90.8% of control) and a PD-L1 monoclonal antibody (77.9% of control), as well as small-molecule inhibitor [86]. Protease-activatable designs (e.g., uPA-dependent PL3) directly address the binding-site barrier by keeping CendR function silent during circulation and perivascular contact, then unmasking it in protease-rich tumor tissue to enable transcytosis-driven spread while limiting off-target deposition [87]. In the eye, iRGD, RPARPAR, and PL3 traverse from choroid through the retinal pigment epithelium to the outer nuclear layer and cut leakage by about fifty percent in a laser induced choroidal neovascularization model [90]. Mechanistic work shows that CendR ligands initiate endocytosis at stable pockets on the vessel wall that function as portals for rapid transvascular and tissue transport of drugs and nanoparticles [91]. Method oriented analyses compare D and L peptide isoforms and outline modifications such as retro inverso and cyclization that influence stability and performance of tumor homing peptides, providing options to enhance CendR carriers. Stage-adaptive engineering with RGD gold nanoparticles shows early, looser ECM favors ~ 7 nm carriers via size dominated transport, whereas late, stroma-dense tumors favor larger particles via multivalent α_v_β_3_ interactions, motivating tuning of size, multivalency, and ligand density across disease stages [88]. Ligand and transcytosis platforms are no longer optional embellishments; they are the decisive levers for deep, uniform delivery in solid tumors. The evidence above broadens the scope from integrins to nucleolin and CXCR4 targets, and from static ligation to trigger-gated entry through glutathione and protease activation.

High-affinity targeting can paradoxically worsen deep penetration by amplifying the binding-site barrier: ligands with strong receptor affinity and/or high multivalency are preferentially captured by the first accessible receptors near vessels, leading to perivascular sequestration and reduced interstitial propagation. In this “affinity penetration paradox,” maximizing binding strength does not necessarily maximize tumor coverage; instead, it can suppress the very trans-tissue transport that CendR programs are intended to enable [92]. Accordingly, effective designs often favor moderated affinity/avidity (lower intrinsic affinity, reduced ligand density, or spacing that avoids premature multivalent locking) to permit traversal beyond the perivascular zone before high-avidity capture. A complementary solution is triggered unmasking: protease-activatable ligands (e.g., uPA-dependent PL3) keep the CendR motif latent during circulation and early vascular contact, then expose it only within the tumor microenvironment, mitigating off-target binding while overcoming the binding-site barrier after extravasation.

The mandate now is design discipline with measurable performance gates. First, the affinity-penetration paradox, commonly described as the binding-site barrier, should be addressed through deliberate control of ligand affinity and avidity. Strategies such as reducing intrinsic affinity, lowering ligand copy number, or optimizing spatial presentation allow carriers to bypass perivascular sequestration and achieve deeper interstitial penetration [93]. Second, hard-wire activation to the tumor by using protease or redox switches that unmask CendR or toxin modules only after extravasation, which lowers off-target exposure while preserving rapid tissue transit. Third, adapt particle size and multivalency to stromal state; small, lightly multivalent constructs should be favored in early, looser matrix, while larger and more multivalent formats can exploit receptor clustering in late, collagen-dense lesions. Fourth, stabilize peptide ligands through cyclization or D-amino acid design without sacrificing receptor engagement, and verify endocytic routing to neuropilin 1 portals with intravital or high-resolution imaging readouts. Fifth, move manufacturing out of the artisanal stage by specifying ligand identity and orientation with orthogonal click chemistry, reporting exact copy number per particle, and linking these metrics to lot-release criteria for receptor binding, transcytosis rate, and penetration depth in standardized tissue models. Finally, couple pharmacology to imaging. Embed traceable surrogates to quantify receptor occupancy, transvascular flux, and intratumoral coverage in real time, then use these data to schedule stage-adaptive dosing and combinations with cytotoxics or immunotherapy. Receptor-guided and transcytosis routes are benchmarked in Table 4. If these requirements are executed with rigor, ligand and transcytosis systems can convert endothelial recognition into reliable cross-barrier transport, extend distribution from rim to core, and deliver durable therapeutic benefit across heterogeneous, stroma-rich tumors.

## Multistage nanodelivery systems

In solid tumors, uniform penetration remains difficult because delivery must traverse sequential barriers, from circulation and vascular entry to interstitial transport, cellular uptake, and on-site release, within a dynamic TME. Multistage systems address this by coupling long-circulation with local transformation and microenvironment priming, converting tumor cues (acidic pH, ROS, enzymes, GSH, hypoxia) into changes in size, surface charge, shell integrity, or ligand exposure. The guiding logic mirrors “long circulation, local activation”: carriers remain stable in blood, then switch at the tumor to deepen diffusion and synchronize payload action [94]. Multistage nanodelivery systems integrate several branches in Fig. 1, combining specific trigger types, carrier transformations and sequential activation steps into a single cascade that spans all penetration levels. To enable cross-platform evaluation beyond qualitative imaging, Table 5 consolidates standardized (or proxy) penetration readouts when reported, including z-stack penetration depth in 3D spheroids (µm), perivascular transport distance in tissue sections (µm), and ROI-/distribution-based indices or ratios, each with explicit comparator groups [95].

**Table 5:** Multistage nanodelivery systems

Platform	Triggers	Carrier/key components	Multistage actions (succinct)	Primary barriers addressed	Penetration model/key readouts in tumors	Component complexity (approx.)	Ref
HA or MMP-2 cleavable coated size-shrinkable system	Enzyme and pH	HA or MMP-2 cleavable shell with CD44 recognition; doxorubicin payload	Long circulation; shell shedding contracts size from about 150 nm to about 50 nm at tumor; pH-guided drug release	Interstitial resistance; cellular uptake	Size contraction and CD44-mediated uptake	Ⅳ: carrier + cleavable shell/HA + targeting (CD44 via HA) + payload	[96]
MOF@poly(ortho ester) core–shell nanocomposite	Acidic pH	MOF core with acid-labile poly(ortho ester) shell; doxorubicin or cisplatin	Blood stability with negative surface; shell loss in TME exposes positive core; nuclear-proximal delivery	Vascular to interstitial transport; cellular entry	3D spheroid z-stack signal: ~ 120 µm; tumor inhibition rate: 31%	Ⅲ: core + responsive shell + payload	[97]
ROS and pH cascade-responsive supramolecular nanoparticles	H_2_O_2_ and low pH	GA-targeted host–guest assembly carrying triptolide	Size decrease from about 181 nm to about 125 nm; zeta potential switches from about − 2.8 mV to about + 15.4 mV; accelerated entry and burst release	Interstitial transport; endo-lysosomal escape	Size: 181.5 → 124.6 nm; ζ: − 2.8 → + 15.4 mV; tumor inhibition rate: 84.14%	Ⅳ: assembly + targeting + trigger cascade + payload	[98]
Hierarchical micelles for curcumin and doxorubicin	pH and enzyme	Biotin-channeled curcumin prodrug micelles with doxorubicin	Stage pH then enzyme response; P-gp inhibition first; doxorubicin release next	CAPIR sequence barriers; multidrug resistance	Spheroid/tissue imaging; standardized penetration	Ⅳ: micelle carrier + prodrug module + 2nd payload + enzyme-responsive step	[99]
“Hairy” molecularly imprinted nanocapsules for sialic acid	Mildly acidic pH and GSH	SA-imprinted capsules with benzoic imine brushes and disulfide crosslinks; 5-fluorouracil model	Brush shedding exposes SA sites and flips surface charge to positive; intracellular GSH degrades shell for rapid release	Transcytosis or active uptake; deep tissue retention	In vivo tumor ROI fluorescence increased by 2 times	Ⅴ: imprinting/recognition + brush + redox crosslinks + payload + pH/GSH switching	[100]
NIR-II AIE luminogen with NO-donor liposomes (Lip-DTI/NO)	NIR light and NO; peroxynitrite cascade	Liposomes with NIR-II AIEgen and SNAP NO donor	Photo-driven O2•- and heat; NO release; ONOO − activation of MMPs; collagen depletion creates a window for penetration; imaging and therapy combined	Stromal collagen; interstitial fluid pressure	Spheroid/tissue imaging; fluorescence increased by 5.7 times	Ⅵ: liposome + photothermal/photodynamic module + NO donor + external energy + cascade (ONOO⁻/MMP) + remodeling readouts	[101]
Paclitaxel-IR780 platform with GSH-responsive release	Photothermal priming and GSH	Paclitaxel conjugate or formulation with IR780; redox-cleavable linker	Photothermal heating increases permeability; intracellular GSH triggers paclitaxel release; chemo-photothermal synergy	Vascular permeability; intracellular release	—	Ⅴ: carrier + payload + photoabsorber + redox linker + external energy	[102]
Nanozyme-assisted sonodynamic therapy	Low-intensity ultrasound and catalytic O2	Hyaluronic acid anchored nanozyme for SDT	Long circulation and targeting; ultrasound triggers SDT; nanozyme converts H_2_O_2_ to O_2_ to relieve hypoxia; macrophage repolarization to M1	Hypoxia; immune suppression	3D tumor spheroids CLSM Z-stack; NR	Ⅳ: carrier + catalytic nanozyme + HA targeting + external energy	[103]
pH and MMP-9 responsive liposomes co-delivering GB1107, BMS1166, enzalutamide (GBE@LP)	Acidic pH and MMP-9	PEGylated liposomes with immuno-modulators	Long circulation; tumor-site release; immune TME remodeling; reversal of T-cell exhaustion	Vascular entry; on-site release; immune barriers	—	Ⅴ: liposome + multi-payload (3 drugs) + dual trigger logic + PEG/stealth	[104]
Hypoxia-reduced charge-switch micelles that trigger transcytosis	Hypoxia and low pH	N-oxide based micelles with CBP decoration; gemcitabine derivative and galunisertib	Stealth during circulation; reduction to tertiary amines switches charge; activation of transcytosis; TGF-β/SMAD stroma remodeling	Deep transport beyond 400 μm; stromal resistance	NR; Perivascular transport distance 407.5 ± 41.2 µm	Ⅴ: micelle + hypoxia-switch chemistry + targeting (CBP) + 2 payloads + remodeling axis	[105]
Acid-induced sphere-to-fiber assemblies	Acidic pH	Dual-drug supramolecular assembly	Long circulation; sphere-to-fiber transition at tumor; prolonged residence and synchronized exposure	Interstitial retention; coordinated payload action	—	Ⅳ: supramolecular carrier + pH shape switch + dual payload + retention design	[106]
Ferritin nanocages with MMP-2/9 cleavable anti-PD-L1 peptide and lysosomal pH release of oxaliplatin	MMP-2/9 and lysosomal acidity	HFn displaying anti-PD-L1 peptide via MMP-cleavable linker; oxaliplatin inside	EPR and TfR1-mediated entry; matrix cleavage exposes anti-PD-L1 function; lysosomal pH releases oxaliplatin	Matrix binding-site barrier; cellular uptake	NR; tumor inhibition rate: 23.7%	Ⅴ: ferritin carrier + peptide checkpoint module + cleavable linker + payload + inherent targeting/entry axis	[107]
Cross-scale modeling for multistage optimization	Computational parameters and TME hydraulics	Models of vascular, interstitial and cellular transport	Predicts size, charge, IFP and diffusion windows; supports stage-aware design and scheduling	Diffusion versus retention; uptake field shaping	**—**	—	[108]

### Transform-switch systems

In three dimensional tumor settings, transformable carriers convert local pH, ROS, enzyme, or GSH cues into size and charge changes that lower extracellular resistance and improve cellular uptake. Enzyme and pH orthogonality can first shed HA or MMP-2 cleavable coats to contract particles from roughly 150 nm to about 50 nm, then trigger pH-guided doxorubicin release under CD44 recognition [96]. Core–shell MOF@poly(ortho ester) structures lose the acid-labile shell in the TME and switch surface potential from negative to positive, supporting nuclear-proximal delivery [97]. In 3D drug-resistant tumor spheroids, this platform showed markedly deeper intratumoral fluorescence, with confocal z-stack signals observable to ~ 120 µm depth (vs largely peripheral signal in the free-DOX control), supporting a penetration advantage under identical imaging settings. Consistent with this, ex vivo organ/tumor fluorescence quantification showed higher tumor-associated signal for the nanoparticle group (tumor ~ 21.0%) while free DOX distributed predominantly to off-target organs (e.g., liver, kidney, lung), providing a second quantitative proxy for more tumor-localized exposure. ROS/pH cascades detach β-cyclodextrin shells, shrink size (~ 181 to ~ 125 nm), and flip *ζ* potential (− 2.8 to + 15.4 mV), which accelerates triptolide entry and burst release at depth [98]. Importantly, efficacy was also benchmarked against matched controls: the tumor inhibition rate reached ~ 84.14% for the GA-targeted cascade system versus ~ 70.88% (non-GA nanoparticle control), ~ 64.87% (free drug), and ~ 22.67% (saline). Hierarchical micelles can stage pH then enzyme responses to liberate curcumin for P-gp inhibition and amplify doxorubicin against multidrug-resistant lesions [99]. Dual pH/GSH micelles combine charge reversal with disulfide cleavage to co-deliver siPD-L1 and indocyanine green, aligning photothermal guidance with checkpoint silencing [24]. And “hairy” molecularly imprinted polymer nanocapsules that shed benzoic imine brushes in acidic TME to expose sialic acid sites, switch charge, and then disulfide degrade for rapid payload release and deep spheroid penetration [100]. Representative multi-interaction designs have demonstrated marked therapeutic impact in hypovascular metastases by integrating organ-selective targeting/charge conversion with catalytic stress, endo/lysosomal escape and antigen capture, thereby enhancing PD-L1 gene silencing and sustained DC/T-cell activation [109]. Quantitatively, confocal depth profiling showed that at 100 µm depth the MIP nanocapsules retained ~ 6 × higher fluorescence than the non-imprinted control (CP), whereas CP signals dropped sharply beyond ~ 40–60 µm, indicating more durable deep-layer delivery rather than surface-only accumulation [98]. In vivo ROI analysis further showed ~ twofold higher tumor-site fluorescence for the MIP nanocapsules versus CP at matched dosing/timepoints, supporting stronger tumor retention as a complementary distribution metric. When viewed side by side, transform-switch platforms in Sect. "Transform-switch systems" also reveal distinct performance profiles (Table 5). pH- or ROS-only cascades, such as GA-targeted supramolecular nanoparticles, achieve modest size reduction and charge inversion that suffice for improved penetration and efficacy in hepatocellular carcinoma, but their activation strongly depends on the presence of intense oxidative stress and acidity [98]. Hierarchical micelles and “hairy” molecularly imprinted nanocapsules integrate recognition and multi-step disassembly, resulting in deeper spheroid penetration and sustained intratumoral retention, yet they require multiple orthogonal linkers and non-trivial synthetic routes [110]. Hypoxia-reduced charge-switch micelles that concurrently trigger transcytosis and stromal remodeling stand out for depth of transport, reaching beyond 400 µm in dense pancreatic tumors and providing robust chemo-immunotherapy synergy, but they also exemplify the highest level of formulation complexity. Overall, systems with more orthogonal triggers and integrated stromal modulation tend to deliver the best combination of penetration and therapeutic benefit, whereas simpler single-trigger designs offer easier translation but more modest gains, highlighting a continuum between performance and manufacturability [111].

Beyond efficacy, the aggressive mechanisms exploited by many transformable platforms also raise non-trivial safety concerns. Enzymatic ECM degradation and broad MMP activation can loosen structural constraints on tumor and stromal cells, and over-depletion of collagen or hyaluronan has been linked to enhanced invasion, metastatic dissemination and, in some settings, vascular complications [112]. ROS-amplifying systems, including photodynamic, sonodynamic and Fenton-like nanoplatforms, deliberately generate high levels of oxidative stress; if spatial confinement fails, excess ROS can injure peritumoral endothelium and fibroblasts, aggravate inflammation or drive secondary fibrosis in normal tissues. Platelet-mimicking coatings and platelet membrane cloaks improve homing and retention, but they also intersect with coagulation pathways and may, in principle, increase the risk of microthrombosis or coagulation abnormalities if not carefully dosed and monitored [113]. Similarly, strategies that recruit or reprogram macrophages must account for the dual roles of tumor-associated macrophages: insufficient control of polarization or trafficking can unintentionally promote immunosuppression, angiogenesis or metastatic niche formation [114]. These liabilities do not negate the value of ROS-, ECM-, platelet- or macrophage-based strategies, but they emphasize that transformable nanomedicines require tight spatiotemporal control (e.g. dual-gated activation, logical “AND” gating and dose optimization), and long-term safety evaluation in immunocompetent models that explicitly monitor metastasis, vascular events and organ damage.

### Priming and remodeling systems

A first pulse primes vasculature and stroma, creating a transient window of lower interstitial fluid pressure and higher permeability, after which the transformed carrier exploits the opened paths. This “prime-first, deliver-second” choreography widens the perfusion window and reduces matrix drag before intracellular action.NIR-II photosensitizer and NO-donor liposomes generate ONOO⁻ in situ, activate MMP-1/2, deplete collagen, increase deposition, and then perform PDT/PTT for tissue-scale penetration in stroma-rich tumors [101]. In this system, MMP-1 and MMP-2 were upregulated by ~ 5.7 × and ~ 5.1 × (vs PBS), while collagen I was reduced to ~ 46% of the PBS group, quantitative remodeling that mechanistically supports the reported penetration gains rather than relying on qualitative imaging alone. GSH-responsive paclitaxel-IR780 platforms use photothermal priming to raise permeability and then rely on redox conditions for intracellular release, yielding chemo-PTT synergy [102]. Nanozyme-assisted sonodynamic formulations repolarize tumor-associated macrophages to M1 and convert H₂O₂ to O₂ to relieve hypoxia, amplifying low-intensity ultrasound therapy and improving distribution. pH and MMP-9 responsive liposomes co-delivering the galectin-3 inhibitor GB1107, the PD-1 inhibitor BMS-1166, and enzalutamide to remodel the immune TME and reverse T-cell exhaustion [103, 104]. Thermo- and hypoxia-adaptive assemblies extend this logic: thermosensitive liposomes pair photothermal heating with HSP90 suppression to overcome thermotolerance [115]; hypoxia-reduced charge-switch micelles achieve transport beyond 400 μm in desmoplastic pancreatic tumor tissue sections and reprogram TGF-β/SMAD stroma [105]; acid-induced sphere-to-fiber transitions prolong residence and synchronize dual-drug exposure [106].

### Recognition-integrated cascades

Cascade designs embed recognition into the delivery sequence so that depth and specificity rise together. Ferritin nanocages use EPR and transferrin receptor 1 for accumulation and entry, then unmask an MMP-2/9 cleavable anti-PD-L1 peptide in the matrix and release oxaliplatin under lysosomal pH, improving chemoimmunotherapy with spatial control [107]. In vivo benchmarking against explicit comparators showed that the OXA and a “αPD-L1 + OXA” combination regimen (~ 51.6% of PBS). Survival was also extended, with median survival improving from 37 days (αPD-L1 + OXA) to 49 days (cascade ferritin system) under the same dosing schedule. Molecularly imprinted nanocapsules leverage sialic-acid recognition for receptor-mediated transcytosis and sustained retention before redox-triggered release [100]. A remotely boosted catalytic antigen-captured nanosponge further exemplifies ‘multiple interactions’ by coupling marginated accumulation with dual-oxide CDT, field-enhanced ROS generation and antigen transport to program dendritic cells, yielding robust suppression of lung metastases when combined with anti-PD-1 therapy [116]. Membrane-disrupting amphiphiles can weaken cell–cell junctions and induce NanoEL-like permeability. In an endothelial monolayer model (bEnd.3), TEER decreased at 6 and 24 h after nanoparticle exposure, and Transwell assays and CLSM confirmed partial leakage across the cell sheet, supporting barrier loosening that can facilitate deeper transport. In vivo, combination regimens that pair structural disruption and immunotherapy further amplify efficacy. For example, mice receiving ONC201@WINA + HFMF plus anti-PD-1 showed the most pronounced survival benefit versus PBS and single-therapy controls, consistent with enhanced T-cell infiltration and improved checkpoint blockade responsiveness [117]. Representative multistage platforms and their evaluation frameworks have been systematically tabulated in Table 5. Multilayer constructs and orthogonal linkers raise manufacturability and reproducibility demands; heterogeneous stimuli risk premature disassembly or under-activation; binding-site barriers and the protein corona can trap carriers near vessels. Modeling indicates that sub-50 nm dimensions favor diffusion, whereas interstitial fluid retention elevates IFP and shapes uptake fields, underscoring the need to co-optimize particle physics with tumor hydraulics [105, 108, 115]. In parallel with computational modeling, PDO-based explants, vascularized organoids and microfluidic tumor-on-a-chip systems can provide intermediate complexity between spheroids and in vivo models, enabling quantitative mapping of penetration profiles under patient-matched stromal and vascular architectures [118]. Recent studies indicate that corona composition is highly context-dependent and can re-mask targeting ligands or pH/redox-sensitive linkers, effectively broadening or shifting activation windows compared with corona-free buffer conditions. Accordingly, multistage and transform-switch platforms should be calibrated in serum-containing media, plasma, or patient-derived matrices, and corona management (stealth coatings or deliberately engineered coronas) should be incorporated into design rules for reliable transformation kinetics in vivo [119]. Priorities are fewer but orthogonal triggers, switch-like activation windows robust to TME variability, and stage-aware tuning of size, charge, and ligand density to match lesion biology. Embedding imageable surrogates for real-time cue verification and adopting click-based, orientation-controlled conjugation with scalable processes offer practical routes to more uniform deep coverage and durable benefit.

## Multistage nanodelivery systems and translational

### Stability and scalability

Transformable and bioinspired formulations face a two-layer stability requirement: (i) *storage and circulation stability* prior to tumor arrival, and (ii) *predictable, trigger-specific conversion* within the lesion. Premature switching can be induced by protein adsorption/corona remodeling, off-target enzymatic cleavage, or heterogeneous pH/redox microdomains, resulting in aggregation, ligand masking, or payload leakage [120]; therefore, stability should be assessed not only in buffer but also under physiologically relevant conditions (serum/plasma), across dilution, freeze–thaw stress, and accelerated storage, using critical quality attributes (CQAs) such as size/PDI, zeta potential, ligand density, encapsulation efficiency/leakage, and trigger-response curves as release criteria [121]. From a scalability perspective, translation favors architectures with fewer unique components and modular chemistries that preserve CQAs during scale-up. For lipid- and polymer-based nanoparticles, controlled and reproducible assembly (e.g., continuous or in-line mixing approaches) combined with in-process analytics can improve batch-to-batch consistency. For bioinspired coatings (cell membranes/extracellular vesicles/cell-based vectors), scalability is often constrained by source variability and purification yield; thus, standardized sourcing, closed processing, sterility/endotoxin control, and functional assays that track key surface markers and penetration phenotypes are essential [122]. Overall, simplifying formulation complexity while retaining robust, lesion-confined activation is the most practical route to achieve both shelf robustness and GMP-compatible manufacturing.

### Clinical translation snapshot of penetration- or TME-modulating nanomedicines

Clinical experience with first-generation approved nanomedicines underscores a recurring pharmacokinetics (PK)-outcome gap in solid tumors: extending circulation time and improving tolerability can be necessary but not sufficient for meaningful survival gains when intratumoral transport remains rate-limiting. Pegylated liposomal doxorubicin (Doxil/Caelyx), for instance, showed similar efficacy endpoints with significantly reduced cardiotoxicity versus conventional doxorubicin in metastatic breast cancer, illustrating that the most consistent clinical value may be toxicity mitigation and exposure smoothing rather than reliably deeper parenchymal penetration[123]. Mechanistically, limited survival translation is commonly associated with heterogeneous EPR/perfusion across patients and lesions, low and variable tumor deposition, perivascular sequestration and binding-site barriers, and carrier/PK designs that do not ensure adequate bioavailable drug release at depth, together with systemic clearance by the mononuclear phagocyte system [124]. Notably, even when a nanoparticle formulation improves outcomes (e.g., nab-paclitaxel plus gemcitabine in metastatic pancreatic cancer), the overall survival (OS) gain can remain modest, reinforcing the need to explicitly target intratumoral distribution and barrier modulation rather than relying on prolonged circulation alone.

These clinical lessons motivate penetration-aware evaluation and patient-/lesion-aware translation frameworks. Against this clinical backdrop, clinical translation remains comparatively sparse and heterogeneous, and many trials do not report standardized intratumoral transport metrics (e.g., penetration distance, coverage, or spatial AUC). Instead, clinical studies typically evaluate safety, systemic pharmacology, and efficacy endpoints that are only indirectly linked to penetration. This gap reflects practical constraints in human tumor sampling, imaging standardization, and the diversity of stromal and vascular phenotypes across indications [122]. To address this translational perspective without overstating clinical maturity, Table 6 provides a concise snapshot of ongoing or completed clinical studies that incorporate penetration-enabling or TME-responsive design principles, including ECM/stroma modulation, pH-responsive or trigger-gated release, redox/ROS-related approaches, and TME-activated immunostimulation. The trials summarized were compiled from three recent review sources, and are presented to clarify the current clinical footprint, trial maturity (phase/status), and the extent to which penetration- or microenvironment-modulating logic has progressed beyond preclinical validation [125].

**Table 6:** Clinical translation snapshot: penetration- or TME-modulating therapies and nanomedicines

Mechanism/ Strategy	Nanomedicine/ Therapy	Phase	NCT ID/ Study	Cancer type	Start/ Completion year	Key outcomes/ Efficacy	Status/ Notes	Reference
ECM/Stroma modulation (HA degradation for penetration)	PEGPH20 + nab-paclitaxel + gemcitabine (HALO-202; HA-high subgroup)	II	NCT01839487	Metastatic PDAC (HA-high)	2013/2018	—	Completed; signal led to phase III	[126]
ECM/Stroma modulation (HA degradation for penetration)	PEGPH20 + AG vs placebo + AG (HALO-301)	III	NCT02715804	Metastatic PDAC (HA-high)	2016/2019	objective response rate(ORR): 47%	Completed; negative	
ECM/Stroma modulation (HA degradation + immunotherapy)	PEGPH20 + avelumab	Early-phase	NCT03481920	PDAC (chemo-resistant)	2018/Terminated	—	Terminated	
General TME-responsive nanomedicine (LNP immunostimulation)	JCXH-211 (self-replicating RNA encoding IL-12 in lipid nanoparticles)	I	NCT05727839	Malignant solid tumors	2023/Ongoing	Good safety profile; antitumor activities observed; increased T and NK cell infiltration	Ongoing; recruiting	[127]
Oxidation/ROS-related nanomedicine (intratumoral redox therapy)	CNSI-Fe(II) (carbon nanoparticle-loaded iron)	I	NCT06048367	Advanced solid tumors	2022/Completed	Safety/tolerability	Completed	
pH-responsive (acid-triggered STING activation)	ONM-501 (pH-sensitive cGAMP-PC7A polymer; ± cemiplimab)	I	NCT06022029	Advanced solid tumors and lymphomas	2023/Ongoing	Clinical trial underway	Ongoing; recruiting	
pH-responsive (sustained TME release via pH-linker)	TranscendIT-101 (TransCon TLR7/8 agonist: resiquimod on PEG microspheres via pH-linker)	I/II	NCT04799054	Locally advanced/metastatic solid tumors	2021/Ongoing	Potent antitumor activity	Active, not recruiting	
pH-responsive cytotoxic nanomedicine (acid-triggered release)	CPC 634 (CriPec® Docetaxel; ~ 65 nm micelle; acid-triggered docetaxel release)	II	NCT03742713	Advanced epithelial ovarian cancer	2018/Completed	—	Completed	
pH-responsive cytotoxic nanomedicine (acid-sensitive linker)	NC-6300 (epirubicin-conjugated micelle; pH-sensitive linker)	I/II	NCT03168061	Advanced solid tumors/soft tissue sarcoma	2017/Ongoing	Promising antitumor activity observed	Ongoing	
pH-responsive cytotoxic nanomedicine (acid-responsive bond)	CriPec® Docetaxel (docetaxel-polymer via acid-responsive bond)	I	NCT02442531	Solid tumors	2015/Completed	Administered safely; cumulative but reversible skin toxicity at high doses	Completed	
Enzyme-responsive (protease/linker logic; targeted NP)	BIND-014 (docetaxel nanoparticles; PSMA-associated targeting; enzyme-responsive linker described in review)	II	NCT01812746	Prostate cancer	2013/Completed	Active and well tolerated; qualitative antitumor	Completed	
Hypoxia/TME modulation (non-nano comparator often discussed in TME context)	Evofosfamide (TH-302) + doxorubicin vs doxorubicin (SARC021)	III	NCT01440088	Advanced soft-tissue sarcoma	2012/2016–2017	No OS benefit; combination not recommended	Completed	[126]
Vascular disruption/perfusion modulation (context trial; non-nano in review)	CA4P (combretastatin A4-phosphate; vascular-disrupting agent)	III	Not specified in review table	HCC/advanced tumors	As summarized	—	Clinical context noted; nano versions mostly preclinical	[128]

### Tumor-type ECM heterogeneity and subtype-dependent penetration mapping

The extracellular matrix (ECM) is highly tumor-type and subtype dependent, which limits the generalizability of any single penetration strategy across indications. Pancreatic ductal adenocarcinoma (PDAC) is often characterized by a dense, desmoplastic stroma enriched in collagen and hyaluronan, associated with elevated stresses/IFP and vessel compression, whereas many breast cancers exhibit prominent collagen remodeling/crosslinking and stiffness gradients, and gliomas/glioblastomas reside in a brain-specific ECM that is comparatively enriched in hyaluronan and proteoglycans with relatively limited fibrillar collagen [129]. Accordingly, ECM/transport barriers should be treated as phenotype-specific design constraints (e.g., HA-rich/desmoplastic vs. collagen-stiff vs. brain-ECM/BBB-constrained tumors), and penetration solutions should be matched to the dominant barrier(s) in a note-of-indication manner rather than assumed to translate uniformly across tumor entities.

To operationalize this concept, we recommend incorporating tumor subtype-dependent penetration mapping into both preclinical benchmarking and early clinical evaluation. Beyond systemic PK and safety, penetration mapping should quantify spatial delivery using standardized metrics (e.g., perivascular distribution, penetration distance/coverage, or spatial AUC) and, when feasible, integrate multimodal readouts such as imaging-based accumulation/heterogeneity, histology-enabled ECM profiling (collagen/HA/proteoglycans), and matched functional endpoints (release/activation markers). Such ECM-informed mapping can support rational patient/lesion stratification, improve interpretability of “negative” trials, and guide the selection of the most appropriate transformable or TME-modulating logic for a given tumor context [130].

## Conclusions and future perspectives

Tumor penetration is fundamentally a transport problem embedded in a heterogeneous and dynamically evolving microenvironment. Across the five strategy families summarized here, namely (i) size and charge transformation, (ii) barrier relief via vascular, extracellular matrix remodeling and interstitial fluid pressure remodeling, (iii) ligand-mediated and CendR or transcytosis mediated transport, (iv) cell based and bioinspired vectors including CPP modules, and (v) multistage cascade systems, the central message is that deep and homogeneous distribution requires coordinated control of vascular access, stromal resistance and on site activation rather than material sophistication alone. Many elegant platforms still stall at the same three failure points: perivascular sequestration, stromal drag and incomplete or misplaced activation [10].

These principles also indicate which strategies merit prioritization for near term translation. Barrier priming approaches that transiently normalize perfusion or decompress stroma are high leverage because they act upstream on dominant transport bottlenecks and can benefit multiple payload classes. When paired with a single, well characterized size or charge switch, they often provide a favorable efficacy versus manufacturability balance. Ligand and CendR or transcytosis systems are likewise high priority when they are designed to overcome binding site barriers through moderated avidity or protease or redox gated exposure. In contrast, highly multistage constructs should be reserved for indications where each added stage has a non redundant role and can be quality controlled. Otherwise, additional stages tend to increase CMC burden faster than they improve patient relevant performance. Cell based and bioinspired carriers remain compelling for specific niches, for example blood–brain barrier delivery, immune cell trafficking and homologous adhesion, but their translational pathway depends as much on standardized sourcing, potency assays and regulatory alignment as on delivery performance [131].

Future research must move beyond uniform delivery paradigms. Emerging evidence highlights that the tumor microenvironment is not merely a passive obstacle but a dynamic, co evolving network shaped by stromal remodeling, immune modulation and neural tumor interactions [132]. Therefore, next-generation nanomedicines should integrate context-aware programming supported by multi-omics guided targeting, organoid-informed and tumor-on-a-chip screening, and patient-matched nanocarrier design. These advanced human-relevant models are particularly valuable for validating deep-penetration claims, benchmarking transport at clinically realistic length scales, and de-risking candidates before first-in-human studies.

Clinical translation readiness should be operationalized with objective gates rather than broad statements. At minimum, candidates should demonstrate (i) limited component count with scalable chemistries and reproducible assembly, (ii) defined critical quality attributes, including size and polydispersity index, ligand copy number and orientation, trigger response curves, release kinetics and stability under storage and in biological fluids, tied to lot release specifications, (iii) penetration relevant performance in human relevant models with standardized spatial metrics, such as coverage, perivascular to core gradients, or spatial area under the curve, rather than qualitative images alone, (iv) evidence that distribution gains translate into bioavailable drug at depth and improved pharmacodynamics, and (v) a safety package aligned with the activation mechanism, including immunogenicity and complement risks for cationic and biomimetic systems [133]. Platforms that are scalable, adaptable and clinically aligned should be evaluated against these gates rather than asserted as a general conclusion [134]. Designing and implementing modular chemistries, such as click based and orientation controlled conjugation, should be treated as a means to meet CMC and critical quality attribute requirements, not as an end point, and should be coupled with imageable surrogates for real time cue verification and modeling guided selection of activation and multivalency thresholds [135].

In summary, the field is moving from smarter materials to transport programmed systems. Designs that target the dominant barrier for a given tumor phenotype, execute a short and verifiable activation sequence, and deliver distribution gains without disproportionate CMC burden are most likely to convert strong preclinical penetration into consistent and patient relevant benefit. In parallel with improving penetration efficacy, transformable and bioinspired nanomedicines should be developed under mechanism-aligned safety constraints, particularly for off-tumor matrix depletion, systemic oxidative stress, and coagulation or immune perturbation. Longitudinal assessment of metastatic spread, thrombotic/hemorrhagic events, and organ toxicities should be incorporated early to de-risk candidates for clinical translation.

## Data Availability

No datasets were generated or analysed during the current study.

## Abbreviations

ADM: Adrenomedullin
AIE: Aggregation-induced emission
AMT: Adsorptive-mediated transcytosis
BBB: Blood–brain barrier
BV: Bevacizumab
CAF: Cancer-associated fibroblast
CD9/CD63/CD81: Tetraspanins (EV markers CD9, CD63, CD81)
CDDP: Cis-diamminedichloroplatinum(II) (cisplatin)
CPP: Cell-penetrating peptide
CRLR: Calcitonin receptor-like receptor
CXCR4: C-X-C chemokine receptor type 4
DMMA: 2,3-Dimethylmaleic anhydride
DOX: Doxorubicin
ECM: Extracellular matrix
EPR: Enhanced permeability and retention
EV: Extracellular vesicle
GSH: Glutathione
HA: Hyaluronic acid
HCC: Hepatocellular carcinoma
IFP: Interstitial fluid pressure
LLC: Lewis lung carcinoma
MMP: Matrix metalloproteinase
MOF: Metal–organic framework
MR: Magnetic resonance
NIR-II: Second near-infrared window
NO: Nitric oxide
NRP1: Neuropilin-1
ORI: Oridonin
PA: Photoacoustic
PD-L1: Programmed death-ligand 1
PROTAC: Proteolysis-targeting chimera
PTT: Photothermal therapy
ROS: Reactive oxygen species
SDT: Sonodynamic therapy
SER: Sensitizer enhancement ratio
T22-PE24: CXCR4-targeted Pseudomonas exotoxin A (PE24) conjugate
TME: Tumor microenvironment
TMZ: Temozolomide
US: Ultrasound
VM: Vasculogenic mimicry
YSA: YSA peptide (EphA2-targeting peptide)
